# Designing flows to enhance ecosystem functioning in heavily altered rivers

**DOI:** 10.1002/eap.2005

**Published:** 2019-10-18

**Authors:** Kevin R. Bestgen, N. LeRoy Poff, Daniel W. Baker, Brian P. Bledsoe, David M. Merritt, Mark Lorie, Gregor T. Auble, John S. Sanderson, Boris C. Kondratieff

**Affiliations:** ^1^ Department of Fish, Wildlife and Conservation Biology and the Graduate Degree Program in Ecology Colorado State University 1474 Campus Delivery Fort Collins Colorado 80523 USA; ^2^ Department of Biology and Graduate Degree Program in Ecology Colorado State University Fort Collins Colorado 80523 USA; ^3^ Institute for Applied Ecology University of Canberra Bruce Australian Capital Territory 2617 Australia; ^4^ Department of Civil and Environmental Engineering Colorado State University Fort Collins Colorado 80523 USA; ^5^ USDA Forest Service, National Stream and Aquatic Ecology Center 2150 Center Ave Fort Collins Colorado 80526 USA; ^6^ Corona Environmental Consulting 357 McCaslin Blvd Louisville Colorado 80027 USA; ^7^ U.S. Geological Survey Fort Collins Science Center 2150 Center Ave. Fort Collins Colorado 80526 USA; ^8^ The Nature Conservancy 2424 Spruce St. Boulder Colorado 80302 USA; ^9^ Department of Bioagricultural Sciences and Pest Management Colorado State University 1177 Campus Delivery Fort Collins Colorado 80523 USA; ^10^ Present address: University of Georgia Athens Georgia 30602 USA

**Keywords:** algae, aquatic insects, channel geomorphology, climate change, designed flow regime, fish, hydrology, modeling, NEPA policy change, probabilistic Bayesian Network model, riparian community, water development

## Abstract

More than a century of dam construction and water development in the western United States has led to extensive ecological alteration of rivers. Growing interest in improving river function is compelling practitioners to consider ecological restoration when managing dams and water extraction. We developed an Ecological Response Model (ERM) for the Cache la Poudre River, northern Colorado, USA, to illuminate effects of current and possible future water management and climate change. We used empirical data and modeled interactions among multiple ecosystem components to capture system‐wide insights not possible with the unintegrated models commonly used in environmental assessments. The ERM results showed additional flow regime modification would further alter the structure and function of Poudre River aquatic and riparian ecosystems due to multiple and interacting stressors. Model predictions illustrated that specific peak flow magnitudes in spring and early summer are critical for substrate mobilization, dynamic channel morphology, and overbank flows, with strong subsequent effects on instream and riparian biota that varied seasonally and spatially, allowing exploration of nuanced management scenarios. Instream biological indicators benefitted from higher and more stable base flows and high peak flows, but stable base flows with low peak flows were only half as effective to increase indicators. Improving base flows while reducing peak flows, as currently proposed for the Cache la Poudre River, would further reduce ecosystem function. Modeling showed that even presently depleted annual flow volumes can achieve substantially different ecological outcomes in designed flow scenarios, while still supporting social demands. Model predictions demonstrated that implementing designed flows in a natural pattern, with attention to base and peak flows, may be needed to preserve or improve ecosystem function of the Poudre River. Improved regulatory policies would include preservation of ecosystem‐level, flow‐related processes and adaptive management when water development projects are considered.

## Introduction

Rivers have been heavily modified on a global scale due to hydrologic alteration by dams and water extraction, leading to extensive ecological change (Nilsson et al. 2005, Dudgeon et al. 2006, Vörösmarty et al. 2010). Ongoing demand for municipal and agricultural water will continue to stress river ecosystems, but those uses are countered by growing interest in restoring rivers to sustainable ecosystem conditions, while still accommodating human needs. Providing water for traditional uses while sustaining ecosystem function poses challenges, particularly in semiarid and arid landscapes where water demand is high (Grafton et al. 2013). Thus, restoration practitioners seek to optimize the functional impact of limited water to maximize ecological outcomes (Yarnell et al. 2015).

River restoration requires understanding linkages between specific flow conditions and ecosystem attributes to provide clear, quantified management targets (Poff and Schmidt 2016, Webb et al. 2017). In heavily altered systems, restoration to a “natural,” pre‐development state is generally not an option, particularly when future climate is uncertain (Moyle 2014, Poff 2018). Alternatively, specifying flows to restore functions that are ecologically important and socially desirable may be possible. So‐called “designer flows” (sensu Acreman et al. 2014) can, in principle, help meet both ecosystem and human needs for water (e.g., Kiernan et al. 2012, Chen and Olden 2017). For heavily appropriated systems with multiple competing users, it is critical to understand how alternative management interventions will affect existing economic and social benefits provided by the river (Northern Colorado Water Conservancy District 2017). It is also important to understand the biophysical processes needed to promote long‐term ecosystem functioning, including dynamic channel features and desirable aquatic and riparian species, which may have different requirements. Appropriate ecosystem modeling that incorporates a variety of future flow conditions is useful for such an evaluation.

The Cache la Poudre River (hereafter, Poudre River) is a southern Rocky Mountains, USA, mountain and plains system in northern Colorado that has been altered by heavy agricultural and urban water use since European settlement in the 1870s. Despite streamflow changes, intensive agricultural and urban land use, and nonnative species establishment, the Poudre River remains a valued amenity both socially and functionally, particularly where it flows through the City of Fort Collins (City). Declining ecological condition of the Poudre River has been documented (City of Fort Collins 2017) but a strong interest has developed among the public and government institutions to restore and promote a dynamic and functioning river that provides amenities. However, extensive dam and diversion infrastructure, proposed additional water development near Fort Collins (U.S. Army Corps of Engineers 2018), and climate change, complicates appropriate management strategies.

Management of arid‐land systems such as the Poudre River requires understanding flow‐ecology relationships (Poff et al. 2010), as well as anticipating future hydrologic change, to illuminate restoration strategies responsive to likely evolution of the river ecosystem. To accomplish this, we first developed a comprehensive, multi‐compartment model informed by empirical data showing how hydrology and other variables (e.g., channel structure, water temperatures, and nutrients) drive important riverine geomorphic processes and associated ecosystem endpoints in the coupled aquatic‐riparian system. Thus, our model differs from other strictly flow‐driven modeling approaches such as ELOHA (Poff et al. 2010), which is effectively a rapid assessment tool useful for multisite comparisons of potential river degradation. Following model development for the current ecosystem, we evaluated how “scenarios” of future hydrologic conditions, ranging from status quo to expanded water development and climate change, may alter the Poudre River ecosystem. We also designed and modeled hypothetical flow regimes that we thought might achieve acceptable ecosystem outcomes under active flow management. Our aim was to produce a scientifically credible and comprehensive analysis to inform the public and assist water managers interested in sustainable management of the Poudre River ecosystem. Here, we detail model development and implementation to identify aspects of an ecologically effective flow regime that might be attainable through active management of water infrastructure, including proposed development in the Poudre River basin. This modeling effort may also inform predictions and management perspectives for other heavily altered river ecosystems in the western United States and elsewhere.

## Methods

### Study site

**Figure 1:**
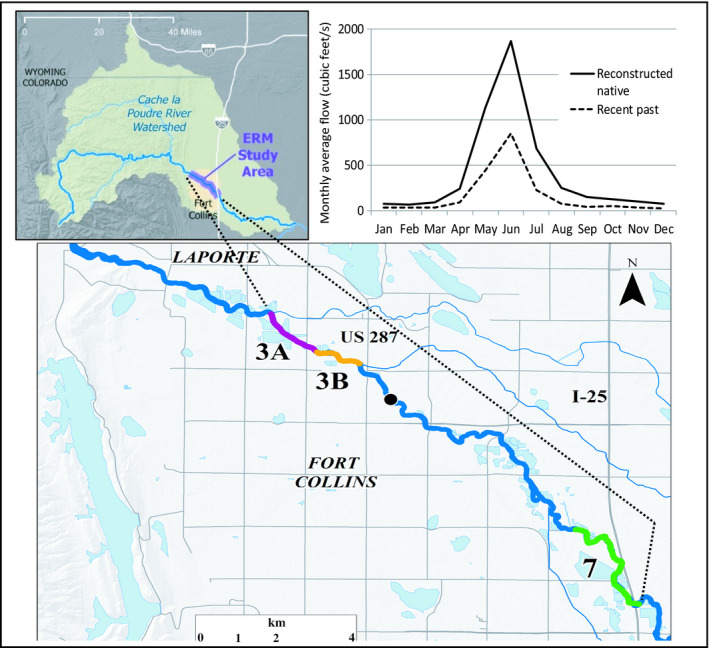
The Ecosystem Response Model study area in the Cache la Poudre River watershed near Fort Collins, Colorado, USA. The Poudre River Basin map (upper left; 1 mile = 1.61 km) shows the study area segment, which is expanded below to show confined, moderately confined, and least confined reaches (3a, 3b, and 7, respectively) from up to downstream. Reduced mean monthly flow of the Poudre River in Fort Collins (water years 1975–2005) for the altered recent past hydrologic scenario (from flow gage measurements, USGS # 06752260; 1 cubic foot/s = 0.03 m^3^/s) is compared to the reconstructed native (pre‐development, modeled flows) flow regime (upper right; Shanahan et al. 2014).

The Poudre River drainage (~2,865 km^2^) originates in high‐elevation mountains (>4,000 m above sea level) west of Fort Collins, Colorado, USA (U.S. Geological Survey [USGS] gage 06752260, Fig. 1). Above 1,900 m elevation, the river is a moderate to high gradient, high‐velocity, cobble‐bottomed stream that supports a trout‐dominated fish community and diverse aquatic insects in orders Ephemeroptera, Plecoptera, and Trichoptera (EPT taxa). In the study area just downstream, the channel meanders through a lower gradient, less confined transition zone between mountains and prairie (~1,600–1,900 m elevation) and supports cool water tolerant trout, native catostomids and cyprinids, and fewer EPT taxa while adding Diptera (Fausch and Bestgen 1997). Native narrowleaf and plains cottonwood (*Populus angustifolia* James and *P. deltoides* W. Bartram ex Marshall, respectively) and their hybrids, willow (*Salix* spp.) and green ash (*Fraxinus pennsylvanica* Marshall), as well as nonnative species crack willow (*Salix fragilis* L.), Siberian elm (*Ulmus pumila* L.), and Russian olive (*Elaeagnus angustifolia* L.), dominate the riparian zone. Gravel, cobble, sand, and silt predominate in this montane‐prairie ecotone. Downstream, the warm‐water Poudre River continues another 60 km to the South Platte River, Missouri–Mississippi River watershed.

**Figure 2:**
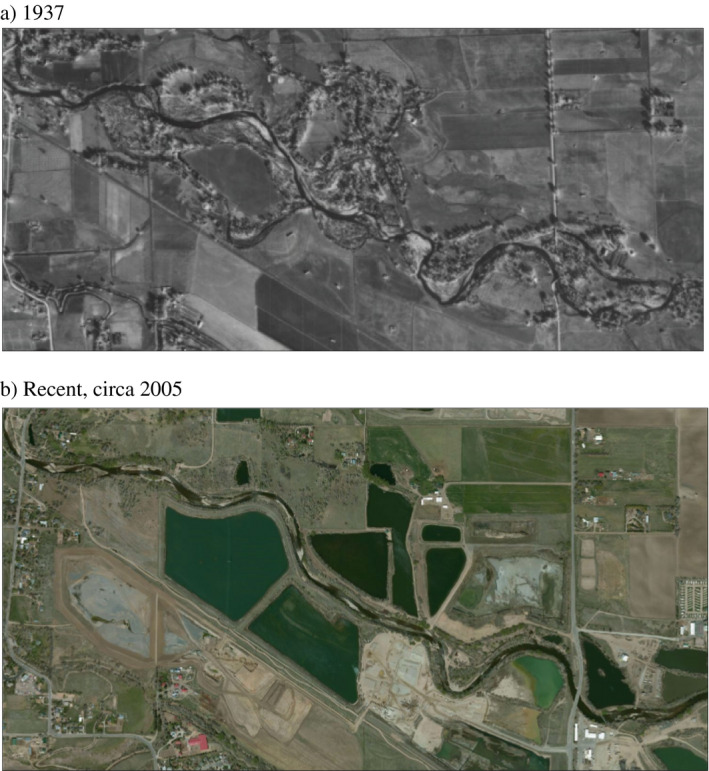
Cache la Poudre River along a section of the ERM study reach, Fort Collins, Colorado, (a) in 1937 and (b) recently (circa 2005). Panel a shows a meandering channel, with a wide, unimpaired zone of channel movement across the floodplain and presence of cottonwood forests of various ages. Panel b depicts the confined channel after nearly a century of land use changes that simplified and straightened the river, reduced channel migration and the associated rejuvenation of riparian habitat, narrowed the riparian zone, and confined the channel with hardened banks and associated pit ponds following gravel extraction.

The 21 km long transition zone reach of the Poudre River, as just described, historically had multiple and sinuous channels and a broad floodplain with oxbows (Fig. 2a). As urbanization and development proceeded, riverbanks were structurally hardened to prevent channel meandering and property destruction during flooding, which resulted in a straighter and mostly confined single‐thread system (Fig. 2b). Native cottonwood and willow dominate the riparian community, although nonnative trees are increasing. Three of eight urban to suburban river corridor sub‐reaches (Fig. 1b) were chosen for modeling because they represented the range of upstream to downstream channel constriction and floodplain connectivity through the 21 km long study area. Reach 3a (confined reach) is highly confined upstream by bank stabilization and has only a few opportunities for floodplain restoration. Just downstream, Reach 3b (moderately confined reach) is partially confined, offering modest restoration opportunity for natural riverine and riparian functions, while downstream Reach 7 (least confined reach) has a mix of armored banks and open floodplain and, potentially, the greatest channel‐floodplain restoration opportunities.

### Conceptual hydrologic calendar

**Figure 3:**
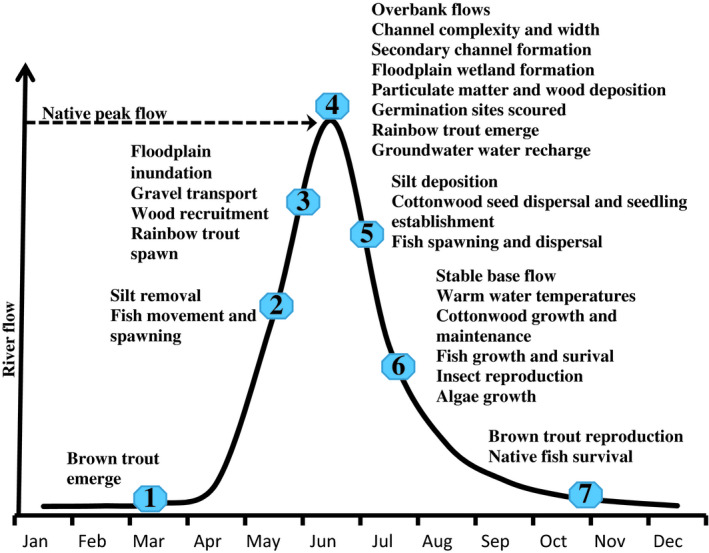
Poudre River hydrology calendar, which conceptually describes flows and timing of functions those flows support to produce physical, chemical, and biological responses.

To illustrate how changes in flows qualitatively affect important geomorphic and biological attributes, we developed a conceptual Poudre River hydrologic calendar (Fig. 3). We developed this model from stream ecology literature (e.g., Allan 1995), regional and Poudre‐River‐specific ecological and geomorphic traits (Fausch and Bestgen 1997, Merritt and Poff 2010, Wohl et al. 2016), as well as from observations and expert judgement based on the authors’ extensive field sampling over the last two or more decades. We adopted this river view after discussions that gravitated from a narrowly focused subset of flow‐biology relationships to a holistic Poudre River ecosystem model useful to predict responses of geomorphic and biological indicators to flow and changes in management. This model reflects our aim of counterbalancing the unintegrated and few species‐specific approaches commonly used in environmental assessments and resource management decision‐making.

Strongly seasonal spring and early summer peak flows foundational to a functioning snowmelt river ecosystem set the physical habitat template for the Poudre River. Increased discharge from high‐elevation snowmelt recruits streamside wood into the channel, mobilizes fine sediments, and scours algae, gravels, and cobbles to create aerated spawning substrates for fishes, including spring‐spawning salmonids. Cool water fishes reproduce and young of spring‐spawning salmonids emerge. High magnitude flow peaks maintain channel width and complexity and sometimes connect the river and floodplain, forming seasonal wetlands of variable extent and duration depending on snowmelt volume. Descending limb flows and associated sediment deposits create germination sites and enhance seedling survival for colonizing plant species (e.g., *Populus* and *Salix*) and enable early life stage fish dispersal to complex, secondary‐channel backwaters. In summer, relatively stable base flows facilitate rapid growth of tree seedlings as well as reproduction and growth of native fishes, trout, and aquatic insects that require cleansed and oxygenated gravel beds. Stable autumn and winter base flows of appropriate magnitude support spawning fish and enhance survival of trout eggs and insects in shallow riffles.

In contrast to the historical conditions portrayed by the hydrologic calendar, the contemporary Poudre River is highly altered (Appendix S1: Tables S1, S2). Extensive water storage infrastructure was developed to supply agriculture and municipal use, aggregate mining and urban development resulted in confined channels, and the many diversion dams upstream of the city (Fig. 1, Appendix S1: Table S1) divert a large proportion of river flow for much of the year. Storage and diversions reduce pre‐development (native) peak and base flows (flows that would occur in the absence of diversions and other management) by 59% and 57%, respectively (Bartholow 2010, Shanahan et al. 2014). These hydrologic changes reduce sediment flushing and contribute to channel simplification thus reducing river amenities including a quality fishery or native riparian corridor (Wohl et al. 2015).

### Model development and structure

Hydrologic alteration induces multiple, linked ecosystem responses, including changes to sediment transport, channel maintenance, and floodplain and wetland inundation, which affect distribution and abundance of in‐channel and riparian biota (Nilsson and Svedmark 2002). Thus, we developed a multi‐compartment Ecosystem Response Model (ERM) to evaluate future trajectories and complex and interacting biophysical functions under various Poudre River flow regimes, using a probabilistic Bayesian Network model. Here, we describe generalities of ERM development; additional details regarding probability tables and relationships used to calculate responses to flows and other variables are in Shanahan et al. (2014), Supporting Information (SI; Data S1) and City of Fort Collins (2019).

**Figure 4:**
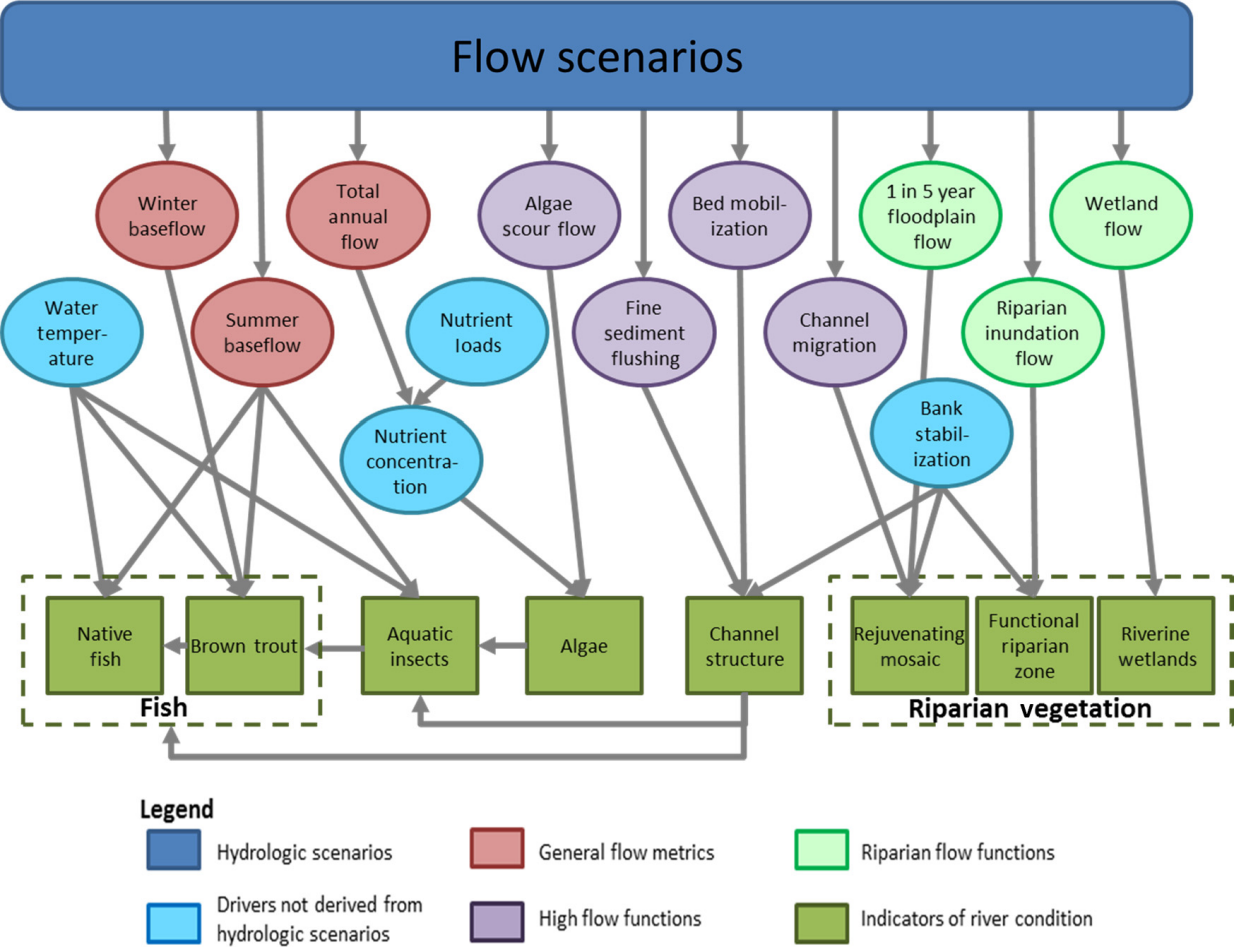
Structure of the Bayesian network for the Poudre River Ecological Response Model (ERM), which links flow regime drivers, including aspects of magnitude, duration, frequency, and variability, to various flow metrics and functions, and their influence on indicators of river condition, the sum of which form ecosystem responses. Arrows between flow metrics and function nodes to indicators of river condition are predictive relationships in the model. Arrows linking indicators of river condition reflect interactions.

The probabilistic ERM network conceptualizes cause‐and‐effect relationships between flow regime, sediment, temperature, and ecological states (Fig. 4). Most relationships are based on conditional probabilities such that effects of one driver on a response will vary depending on other driver variables. Use of conditional probabilities leads to complex model parameterization but allows for incorporation of many information types to produce predictions about physical, chemical, and biological resources, and interactions among them. Because hydrology is a known master driver of physical and ecological conditions in streams (Poff et al. 2016, 1997), the ERM can be used to predict outcomes under various conditions including native flows, present altered flows, and future regimes resulting from additional water storage or climate change. The ERM incorporated major ecosystem components and interactions and retained advantages of a Bayesian Network approach (Uusitalo 2007) including (1) integration of various ecosystem functions typically evaluated as independent variables, (2) incorporation of various data types ranging from quantitative empirical analyses to qualitative expert judgment, (3) explicit quantification and incorporation of uncertainty, and (4) flexibility to test an array of scenarios.

Indicators were formulated using combinations of quantitative channel hydraulics, empirical flow‐ecology relationships based on continuous or categorical responses, and interacting effects of flow mediated through various combinations of base and peak flow, temperature, nutrients, and bed stability. Indicators included in the ERM (see Appendix S1: Table S3) were: (1) channel structure (substrate and channel geometry template for physical and ecological processes), (2) algae (basal food web resource, but unaesthetic and detrimental when excessive), (3) aquatic insects (species composition and abundance indicates flow regulation, water quality, and is a critical food web link), (4) native fish (indicates channel condition and flow regulation effects), (5) trout (mainly nonnative brown trout (*Salmo trutta* L.), which have high angler value and are a sensitive indicator of thermal and hydrologic regimes), (6) rejuvenating mosaic forest (width of multistage riparian forest with species adapted to disturbance), (7) functional riparian zone (river‐connected area that supports biogeochemical processing, flood peak attenuation, sediment deposition, episodic aquatic habitat, and a productive vegetative community), and (8) riparian wetland (floodplain area inundated with sufficient frequency and duration to support wetland plants). Indicators were grouped into three types, based mainly on the amount of quantitative data available to describe them. The first group, for which quantitative data were available, included channel structure and three indicators of riparian condition, for each of the three separate river reaches. Because quality and quantity of stream habitat are determined by the interaction between flow and the structure of the river channel, the effects of flow changes on the ecosystem must be considered in the context of the current channel structure and its variability along the river (Brewer et al. 2016, McManamay et al. 2016). To quantify the effects of channel structure and associated moderate to high flows on indicators in the ERM (i.e., algae, native fish, trout, aquatic insects, and three riparian vegetation indicators), shear stress and effective discharge analyses were performed at representative locations in each of the three reaches modeled along the Fort Collins river corridor. Hydraulic modeling identified discharges at which critical thresholds of shear stress, associated with riverbed flushing and bed and channel mobilization, were met, based on flow characteristics, channel geometry, and substrate composition in each reach (details in Shanahan et al. 2014; the full channel structure model data and a detailed narrative is in SI, and Data S1; the full Excel spreadsheet is also available from the senior author upon request). An annual high flow pulse capable of flushing surface deposits of fine sediment was assumed needed to ensure ecological functioning, while widespread mobilization of the coarse river bed sediments had a longer, two‐year average return interval based on the current management infrastructure, and on interannual flow variability including multi‐year dry periods. Descriptions and data sources for cross‐sectional geometry were used to perform shear stress and effective discharge analyses, discharge–shear‐stress rating curves, the HEC‐RAS model (U.S. Army Corps of Engineers 2009), hydraulic model median grain size (*d*_50_), and flow records for each of the three reaches, as described in Shanahan et al. (2014), SI (Data S1) and City of Fort Collins (2019).

Geospatial probability modeling was used to determine floodplain area available for the three riparian indicator responses. Reach‐specific empirical models related flood flow inundation to riparian forest species and functional group composition. These relationships used detailed riparian plant distributions (Shanahan 2009) and measured presence of the rejuvenating mosaic, functional riparian zone, and riverine wetlands, and were modeled as a function of exceedance probability from a 30‐yr flow record (USGS streamflow gages) using logistic regression. Compared to the other two riparian indicators that mainly require floodplain inundation, the rejuvenating mosaic requires higher shear stresses to induce channel migration and to disturb and scour floodplain germination sites for seeds. Exceedance probability was mapped using local rating curves developed with HEC‐RAS 1‐D hydraulic models (U.S. Army Corps of Engineers 2009), a 1‐m^2^ digital elevation model, and river flow duration curves. Reconstructed historical flows and future climate change and water development scenarios were used to recalculate and reproject future exceedance probabilities and corresponding distributions and area of vegetation, which informed probabilistic model parameters.

The trout indicator was the sole member of the second indicator group, which was based on an empirical flow‐ecology relationship augmented with expert judgement. The trout indicator was based in part on field sampling that related abundance of young brown trout captured in autumn samples (*n* = 16 yr) as a function of the river flow level in the previous winter when eggs were incubating and hatching. This relationship indicated that higher winter flows of about 1 m^3^/s, for example, had a relatively high 0.67 probability of producing a larger number (>20) of young trout per year, while low flows < 0.28 m^3^/s had an 80% probability of producing 5 or fewer trout; intermediate flows produced an intermediate number of young trout. The empirical relationships between winter flow categories and young trout abundance were used to describe the probabilities of having a trout fishery in one of four categories, or states (−−,−, 0, +) that reflect the number of age classes present, their abundance, and reproductive success (present state is between − and 0). Several other factors also influenced this indicator (see Fig. 4), and these were assigned independent probabilities (by expert judgement) to place trout into one of the four states in a process similar to that described below for qualitative indicators (see SI; Data S1; City of Fort Collins 2019). We also weighted driving variables for each indicator in the ERM according to their relative importance. Using trout as an example, weights for winter baseflow, summer baseflow and temperature, and channel structure were relatively high and equal (0.27 each, total of 0.81), reflecting that habitat and temperatures are relatively more important, while invertebrates received a lower relative weight (0.19), reflecting that trout can likely obtain ample food even in a relatively degraded system. We also detail the full progression of the trout indicator, including several interacting flow‐related metrics and probability tables, across the range of environmental drivers to demonstrate how we arrived at the final reach‐specific indicator states (see SI; Data S1).

Expert judgment was used to assign flow‐based or other probabilities to a third group of indicators, algae, aquatic insects, and native fish, in the absence of direct flow‐ecology relationships. For example, aquatic insects in each reach were assigned to one of three states: + (many EPT, including insects with 2‐yr life cycles), 0 (mostly EPT but univoltine and reduced abundance) and − (some EPT but many tolerant taxa as well). Insect community probability state was a function of three designated drivers (see Fig. 4) of community composition and abundance: (1) channel structure (a function of fine sediment flushing, bed mobilization and bank stabilization), (2) summer base flow magnitude and water temperature above or below 23°C as one combined variable, and (3) algae production (a function of nutrient concentration and scouring flow). For example, a clean and diverse streambed had respective probabilities of producing aquatic insect states −/0/+ of 0.0/0.5/0.5. Note total probability sums to 1.0 across the three states. Adequate summer baseflow combined with cool temperatures generated probabilities for aquatic insect states −/0/+ of 0.0/0.5/0.5. For algae, where future abundance was “about the same as today” insect states −/0/+ were assigned probabilities of 0/1/0. Thus, in a river reach, under a given flow scenario that generates a clean and diverse streambed, adequate and cool baseflow, and about the same amount of algae as today, the conditional probability of an aquatic insect state of 0 is calculated from the product of the probabilities of the three controlling variables, i.e., 0.5 × 0.5 × 1 = 0.25. Similar reasoning was followed for other response variables lacking suitable empirical monitoring data. For example, probability tables for the impacts of nutrient enrichment (total nitrogen and dissolved phosphorus) and scouring flows on algal biomass were based on general observations of experts in recent years to generate states of − (less than today), 0 (about the same as today), and + (more than today). Native fish states (−−,−, 0, +) were based on expected species richness, abundance, and life stage diversity in response to summer baseflow, temperature, trout predation, aquatic insects, and channel structure (see Shanahan et al. 2014 and SI [Data S1] for further details). Our fish species richness metrics were tailored to the naturally depauperate local assemblage and reduced species richness due to extirpation of specialists more sensitive to flow alterations (e.g., gravel‐spawning nest builders, Fausch and Bestgen 1997), but could be easily altered for other geographic areas where fish species richness is higher.

Use of expert judgement, based on research experience and published ecological and hydro‐geomorphic principles, is well‐established in modeling and decision analysis (von Winterfeldt and Edwards 1986, Otway and von Winterfeldt 1992). Our main effort to reduce uncertainties associated with expert judgement was to assign conservative conditional probabilities, such that only stressor levels in the highest category were coded to cause ecological impairment. This conservatism may lead to less variation in the absolute expected values of each indicator, but the relative differences across the flow scenarios remained robust. While we specified prior distributions for all parameter interactions, we currently lack sufficient empirical data across all flow scenarios and indicators to refine prior distributions. Hence, we proceeded by specifying network linkages (Fig. 4), computing prior distributions from available data, and comparing results for a single flow scenario (recent past) against other scenarios of interest.

The ERM model uses Structural Modeling, Inference, and Learning Engine software running in GeNIe (Graphical Network Interface; Decision Systems Laboratory 2014) and computes conditional probabilities for input data using the general form

$$P\left(A_{i}|B\right)\;=\;\frac{P\left(B|A_{i}\right)P\left(A_{i}\right)}{P(B)}\;=\;\frac{P\left(B|A_{i}\right)P\left(A_{i}\right)}{\sum_{i=1}^{n}P\left(B|A_{i}\right)P\left(A_{i}\right)}$$

where *A* and *B* are possible outcomes and *P*(*A*_*i*_ *|B*) is the conditional probability of *A*_*i*_ given *B*. The eight ERM indicators (model output) measure aspects of ecosystem function and condition and include variables that have regulatory implications, such as Clean Water Act aquatic life criteria, nutrient thresholds, and water temperatures, and biological indicators valued by the community.

Linkages that determined indicator condition were mapped in the final Bayesian network (Fig. 4). Hydrologic drivers including flow magnitude, duration, and frequency influenced physical processes and ecological states directly and interactively and those were altered to create flow regime “scenarios.” Flow attributes had both direct and interacting effects on indicator condition. For example, peak flow conditions directly affected algae via scouring, and channel structure via sediment flushing and bed mobilization. In contrast, aquatic insects, native fish, and trout indicators had only interacting links to peak flow attributes, via changes in channel structure, because direct relationships were not available from existing data or reliably inferred from expert judgement. Although hydrology was the primary driver of ecosystem responses, other important factors were also incorporated including water temperature, nutrients and water chemistry, and bank stabilization interacting with flows (Fausch and Bestgen 1997, Dudgeon et al. 2006, Poff 2018).

### Hydrologic scenarios

**Figure 5:**
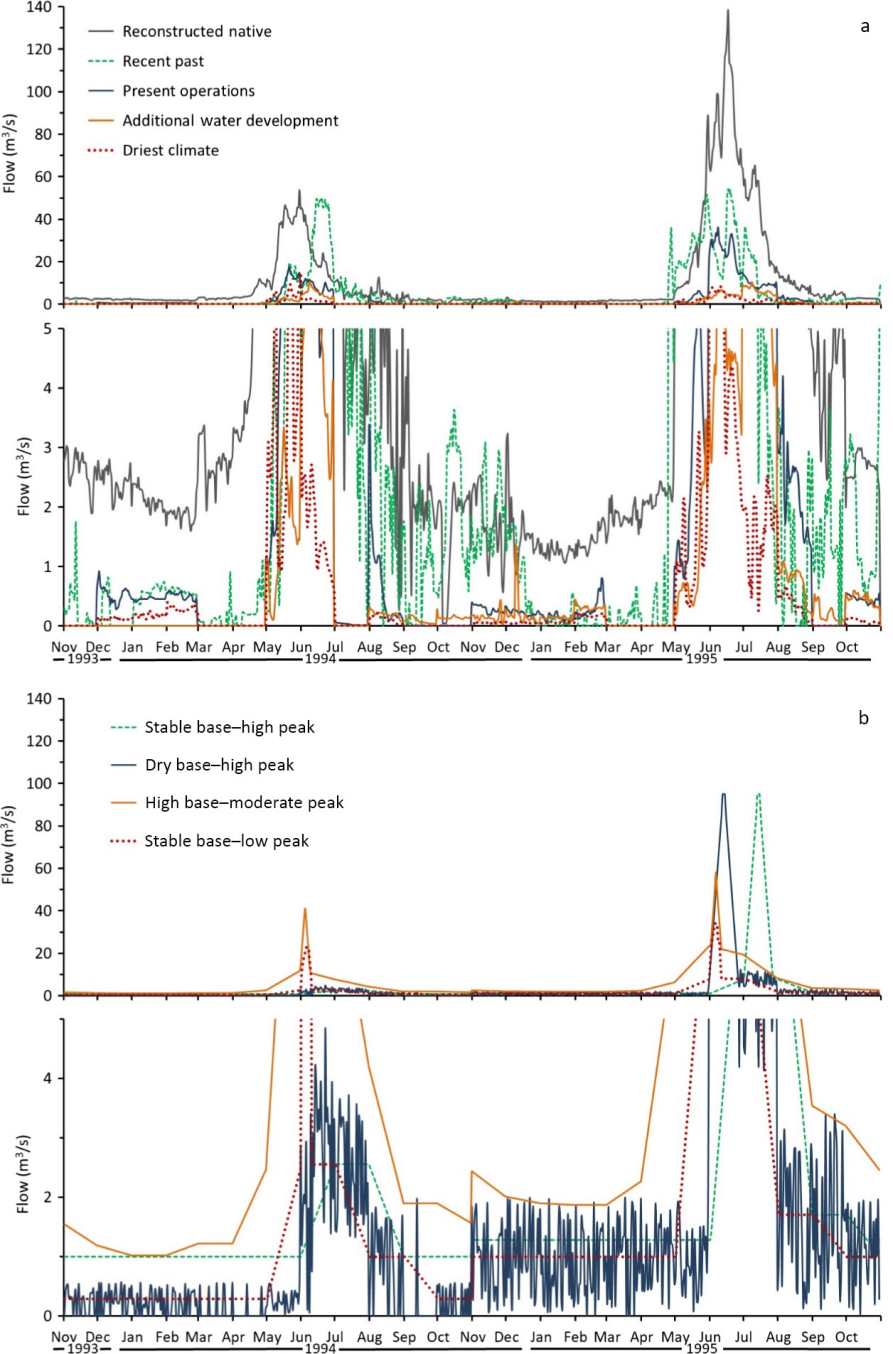
Dry and wet year hydrographs for the Cache la Poudre River, Fort Collins, Colorado, showing differences in peak (upper) and base (lower panel, expanded for detail) flows for (a) five historical or future flow scenarios and (b) four designed flow scenarios. All are modeled flow scenarios with the exception of the recent past, which is from gage data (U.S. Geological Survey # 06752260).

After finalizing the ERM structure, we developed nine hydrologic scenarios as model inputs (Fig. 5; Appendix S1: Table S4). Scenarios characterized their effects on the Poudre River ecosystem (e.g., peak flow frequency, low flow duration) and spanned a spectrum of past to future conditions including

1. three historical scenarios that included historic unaltered regimes (reconstructed native), recent‐past altered flows (recent past), and present, continuing flow alteration (present operations;)
2. two future scenarios with reduced water availability due to additional development (additional water development) or climate change (driest climate); and
3. four designed hydrologic scenarios with combinations of base flow magnitude and consistency, and peak flow magnitude, duration, and frequency to achieve specific ecosystem goals. These we referred to as stable base–low peak, high base–moderate peak, dry base–high peak, and stable base–high peak.

#### Historical and future hydrologic scenario development

Hydrologic scenarios were based on gage records, diversion withdrawal data, and outputs from models used by city planners and regional water managers. All historical and future scenarios were founded on the recent past scenario, a spatially discretized record of gaged discharges across the study reach. Native and present operations scenarios remove (or add) the effect of existing reservoir and diversion operations in the Poudre River drainage. Together, these models and streamflow gages produced time series of simulated flow at a daily time step (Fig. 5; Appendix S1: Tables S2, S3). To incorporate climate change impacts, the present operations scenario was modified using predictions from global climate circulation models (Diansky and Volodin 2002) and the Bias Corrected Spatially Downscaled [BCSD], Coupled Model Intercomparison project phase 3 archive (CMIP3, collectively the BCSD‐CMIP3) that describes climate‐changed hydrologic scenarios for the western United States (Gangopadhyay et al. 2011, U.S. Bureau of Reclamation 2011). Downscaled hydrology data are monthly time series predictions of unit runoff for each circulation model for one‐eighth degree (12 × 12 km) latitude‐longitude grid cells. Runoff calculations used the CMIP3 scenario with the lowest projected runoff in 2050 (inmcm3_0.1.sresb1) for the grid cell that most overlapped the Poudre River basin, and was the basis for our plausible driest climate scenario. To create the hydrology time series, we first computed the monthly ratio of average runoff under the driest climate scenario to average runoff under current baseline conditions. These ratios were then multiplied by the present operations daily flows to estimate the driest climate hydrologic time series of daily flows used with the ERM.

#### Designed flow scenario development

The designed flow scenarios were developed as potential guidelines for water managers with the goal of improving the Poudre River flow regime to achieve certain social‐ecological outcomes (Acreman et al. 2014). Designed flow scenarios have combinations of functional characteristics (e.g., Yarnell et al. 2015) that include base flow magnitude and consistency, and peak flow magnitude and duration. Sufficient base flow magnitude supports habitat for fish and aquatic insects, and influences water temperature and nutrient levels, while flow consistency reduces variation due to high diversion extraction or low reservoir releases that presently create disconnected pools and dry reaches detrimental to aquatic life. Although highest magnitude flows depend largely on snowpack levels, proposed water projects would store additional peak flows and further reduce their magnitude and duration, allowing for the possibility of designed flows to achieve downstream ecological targets if reservoir and diversion operators let flows bypass infrastructure. Designed scenarios (e.g., stable base–high peak) also included ascending and descending limb flow rates of change of about 7.1 m^3^·s^−1^·d^−1^ during the peak runoff period (e.g., Yarnell et al. 2019, 2010, City of Fort Collins 2019); direct effects of limb flows are presumed important but were not modeled. We show two consecutive years of the modeled Poudre River hydrographs for all scenarios (Fig. 5), in consecutive dry (1994) and wet (1995) years, to illustrate differences in base and peak flow magnitude, timing, and variability, among years when snowmelt runoff magnitude differed. Using the ERM relationships between flow and various indicators of river condition, we predicted effects of the four hypothetical designed flow scenarios on Poudre River ecosystem attributes using the same technique as for historical and future flow scenarios.

**Figure 6:**
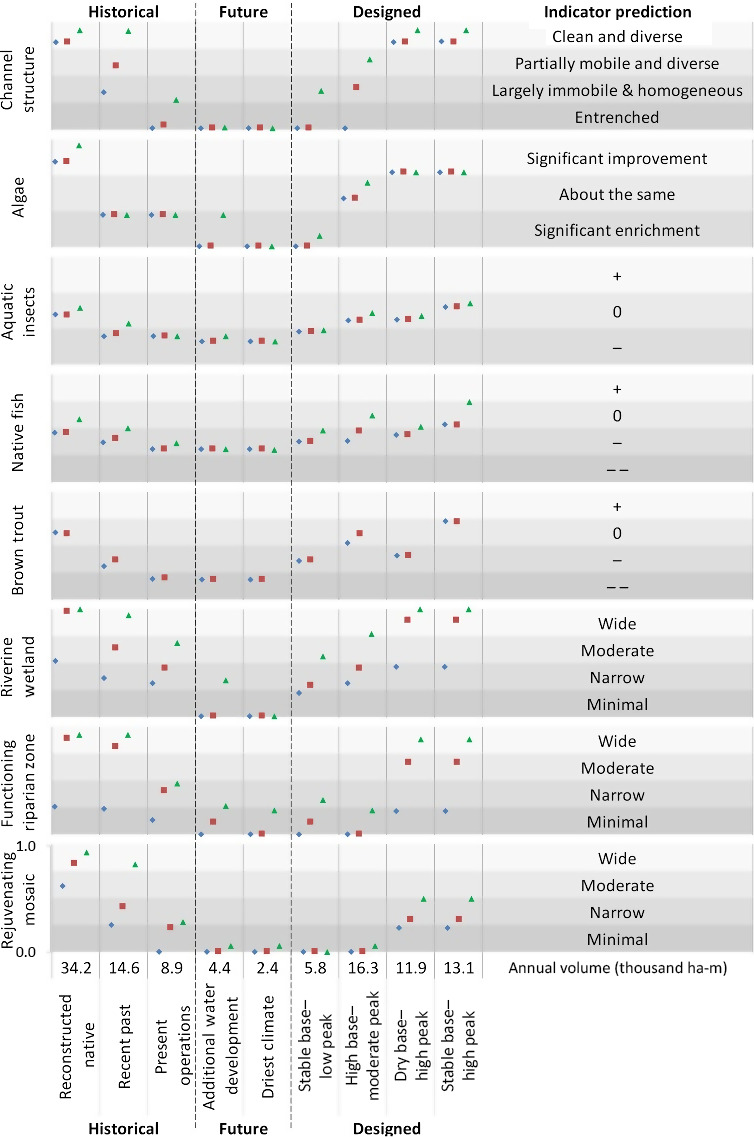
Indicator predictions for three historical, two future, and four designed hydrologic scenarios for eight indicators of river condition in each of three Poudre River reaches. Each indicator is scaled from 0 to 1, with the four different gray‐shaded rows for each indicator showing quartiles of change. From up to downstream, blue diamonds are for the confined reach, red squares for the moderately confined reach, and green triangles for the least confined reach. The annual volume of flow (ha‐m) required to achieve each Hydrologic Scenario is portrayed at the bottom of each results column. Scores for river condition indicators for aquatic insects (+, 0, −) and fish (+, 0, −, −−) are arrayed from lowest to highest. No trout scores are presented for the downstream, least confined reach because water was warm, and few trout were present.

For each of the three reaches evaluated by the ERM, the ecological response of the eight river indicators under nine hydrologic scenarios was computed as a probability distribution scaled from lower (0) to higher (1) functioning. Each distribution is portrayed as a single mean value, which simplifies data presentation (Table 1; details in Shanahan et al. 2014 and SI). Indicator scores were then plotted (Fig. 6) on a probability scale (0–1) with associated qualitative predictions of condition from lowest (0) to highest (1). For example, channel structure scores were assigned to quartiles of the scale that ranged from an entrenched condition (lowest, score of 0–0.25) to a clean and diverse condition (highest, score 0.76–1). Native fish and trout scores from lowest to highest were assigned relative predictions in four ranked classes (−−,−, 0, +) and lowest to highest riparian indicator scores had relative predictions from minimal to wide areas of inundation, respectively. Indicators with only three categories were similarly assigned, where, for example, aquatic insect predictions ranged from −− (lowest condition, score of 0–0.33) to + (highest condition, score 0.67–1.0). Algae scores represented conditions that were significantly enriched and worse than present conditions (lowest, 0–0.33), similar to current conditions (0.34–0.66), or were significantly improved from present conditions (highest, 0.67–1.0). Differences in indicator scores are appropriately interpreted between flow scenarios in comparative rather than absolute terms as 0–1 scales for each indicator varied with input data and assumptions for each prior distribution.

**Table 1:** Index of Poudre River condition for eight indicators in three different river reaches (3a = confined, 3b = moderately confined, 7 = least confined) under nine different hydrologic scenarios

Indicator and reach	Reconstructed native	Recent past	Present operations	Flow scenario		Stable base–low peak	High base–moderate peak	Dry base–high peak	Stable base–high peak
				Additional water development	Driest climate				
Channel structure									
3a	0.80	0.33	0	0	0	0	0	0.80	0.81
3b	0.80	0.58	0.03	0	0	0	0.38	0.80	0.80
7	0.91	0.91	0.26	0	0	0.35	0.64	0.91	0.91
Algae									
3a	0.80	0.30	0.30	0	0	0	0.45	0.70	0.70
3b	0.80	0.30	0.30	0	0	0	0.45	0.70	0.70
7	0.95	0.30	0.30	0.30	0	0.10	0.60	0.70	0.70
Aquatic insects									
3a	0.46	0.26	0.26	0.21	0.21	0.30	0.41	0.41	0.53
3b	0.46	0.28	0.26	0.21	0.21	0.30	0.41	0.41	0.53
7	0.53	0.38	0.26	0.26	0.21	0.32	0.48	0.45	0.57
Native fish									
3a	0.45	0.37	0.30	0.30	0.30	0.37	0.38	0.43	0.53
3b	0.45	0.40	0.30	0.30	0.30	0.37	0.47	0.43	0.53
7	0.58	0.50	0.36	0.30	0.29	0.47	0.62	0.51	0.75
Trout									
3a	0.61	0.30	0.18	0.18	0.18	0.35	0.52	0.40	0.72
3b	0.60	0.35	0.19	0.18	0.18	0.35	0.60	0.40	0.71
Rejuvenating mosaic forest									
3a	0.62	0.26	0	0	0	0	0	0.23	0.23
3b	0.83	0.43	0.23	0	0	0	0	0.30	0.30
7	0.94	0.83	0.29	0.06	0.06	0	0.06	0.50	0.50
Functional riparian zone									
3a	0.25	0.23	0.13	0	0	0	0	0.21	0.21
3b	0.90	0.82	0.41	0.11	0	0.11	0	0.67	0.67
7	0.93	0.93	0.48	0.27	0.22	0.32	0.22	0.89	0.89
Riparian wetland width									
3a	0.51	0.36	0.30	0	0	0.21	0.30	0.46	0.46
3b	0.98	0.63	0.44	0	0	0.28	0.44	0.89	0.89
7	1	0.94	0.68	0.33	0	0.55	0.77	1	1

## Results

Modeling showed indicator variable response patterns typical of many flow‐regulated systems, but it also revealed lesser‐known interactions instructive for ecological understanding and management that varied spatially. Indicator scores were generally highest under the reconstructed native flow regime followed by the two designed flow scenarios with high peaks and the Recent Past regime in the least confined downstream reach (Fig. 6, Table 1). Indicator responses were lowest under future flow scenarios (additional water development or dry climate) in the confined reach. Present operations scenario scores were generally low.

Channel structure and the three Riparian zone indicator response scores were most sensitive (variable) to the array of flow scenarios. Low or zero scores resulted when only low magnitude peak flows were available (e.g., two future scenarios) but channel structure responded strongly to high magnitude flows because key shear stress levels were exceeded (e.g., reconstructed native, two designed flows with high peaks). Among instream biota, algae and trout were most sensitive to flow, responding negatively in the absence of high flows and subsequent impaired channel structure, and positively to presence of higher base flows, especially in winter, and cooler water temperatures in summer. Aquatic insect and native fish scores were the least sensitive to various scenarios because assigned probabilities for various effects were conservatively estimated, mainly because few specific links to flows and other drivers were apparent (Shanahan et al. 2014). Details for indicator responses to flow scenarios are below.

### Channel condition

Channel structure scores declined through the progression from Historical to Future hydrologic scenarios, due to declining peak flows and increased channel simplification, a pattern generally similar for other indicators. Highest channel structure scores (0.80–0.91) under reconstructed native and some designed scenarios resulted from high magnitude flows for a minimum of three consecutive days that provided sediment flushing, coarse substrate mobilization, channel migration, and increased geomorphic complexity. Alternatively, channel structure score was 0 in high base–moderate peak, additional water development, and driest climate scenarios in confined and moderately confined reaches because flow magnitude was inadequate to mobilize substrate and halt channel simplification.

Flows required for substantive geomorphic work varied spatially along the river corridor. Increasing channel structure scores from upstream confined and moderately confined reaches to the downstream least confined reach reflected increased downstream channel migration and complexity. Increased downstream geomorphic work can be achieved, despite identical simulated river flows, because median sediment size decreased more rapidly than channel gradient from upstream to downstream, so the same peak flow magnitudes increased channel structure scores more downstream.

### Instream biota

Algae indicator scores were also highest under reconstructed native and designed hydrologic scenarios with high peak flows (score range 0.70–0.95) but lowest in confined reaches with low peak flows because substrate mobilization and scour were minimal. Identical recent past and present operations scores resulted because flow thresholds that altered channel structure were not achieved.

Aquatic insect scores were highest (0.46–0.57) in high peak and higher base flow scenarios (reconstructed native, stable base–high peak) because those conditions increased taxa richness, life history diversity, and abundance and were lower in confined reaches with low peak flows and low or variable base flows. Native fish indicator scores were higher (0.38–0.75) in scenarios with higher peak flows and consistent base flows (reconstructed native and designed scenarios except stable base–low peak) due to higher taxa richness, life stage diversity, abundance, and channel‐structure‐related habitat diversity, attributes that were reduced in low peak or variable base flow scenarios. Reasons for reduced score ranges over all flow scenarios and reaches for aquatic insects and native fish were discussed above. Native fish scores in the least confined reach were consistently higher, regardless of hydrologic condition, reflecting greater habitat availability and low abundance of predaceous trout in that warmer reach.

Trout reproduction, abundance, and age‐class diversity varied with summer and winter base flow levels, summer water temperatures (higher in low flows), aquatic insect abundance, and channel structure. Thus, highest trout scores (0.40—0.72) resulted from higher peak and consistent base flow scenarios (reconstructed native, high base‐moderate peak, and stable base‐high peak), which was supported by empirical data that linked trout reproductive success with higher winter base flows. Conversely, trout were negatively affected by low base flows in summer (reduced survival) and winter (reduced reproduction), and elevated summer water temperatures that may reduce dissolved oxygen levels. Effects of lower winter base flows are evident by comparing the dry base‐high peak score (0.40) to other designed scenarios with higher base and higher peak flows (score range 0.52–0.72).

### Riparian zone

Riparian forests responded positively to high peak flows that saturated soils, mobilized sediment, and created channel movement, and they responded negatively to low flows and bank armoring, especially in confined reaches. Among historical flow regimes, reconstructed native and, to a lesser extent, recent past scenarios elicited the strongest positive response by the rejuvenating mosaic indicator, particularly in the least confined reach (0.94 and 0.83, respectively). Designed hydrologic scenarios with high peak flows showed the greatest improvement over those with moderate or lower peaks. Native riparian tree recruitment was negligible with low peak flows (score range 0.00–0.29) because floodplain connections rarely occurred, even in the least confined reach.

Scenarios with high peak flows (reconstructed native, recent past) produced the highest functional riparian zone scores, especially in the least confined reach (scores = 0.93), similar to riparian wetland scores (0.94–1.00). Wetland development was limited in channel‐confined reaches under most flow scenarios (confined reach = 0.00–0.51) because high, steep banks and channel entrenchment prevented river–floodplain connections. Similar to the functional riparian zone, wetlands would increase if bank height were reduced and banks were set back and sloped to allow greater river–floodplain connection and a more continuous moisture gradient. Rejuvenating mosaic scores were lower than the other two riparian vegetation scores under the same flow and reach conditions because flow magnitudes and velocities were insufficient to disturb and scour surfaces needed for seed germination sites.

Annual flow volume required to implement the nine ERM flow scenarios varied widely. For example, annual discharge volume in the reconstructed native scenario was more than twice as high (34,246 ha‐m; 278,000 acre‐feet, Appendix S1: Table S2) as other scenarios and up to 14× greater than low peak flow scenarios, regardless of base flow characteristics. Notably, when compared with the reconstructed native or recent past scenarios, the stable base–high peak scenario produced comparable or higher indicator scores for most metrics with substantially less water (13,117 ha‐m; 106,000 acre‐feet, Appendix S1: Table S2). Reach differences for indicators reflected prevalence of overbank flooding, or, of differences in channel structure rather than flows, which were identical across reaches.

All indicators were sensitive to changes in assumptions of driving variables; those with linear or continuous responses were relatively more sensitive than categorical driving variables. For example, increased flows and shear stress caused channel structure change, especially when thresholds for bed particle mobility were exceeded. Channel structure changes then cascade interactively through most instream biological indicators. Categorical variables were less sensitive to flow changes, unless they resulted in response category changes, indicating that additional quantitative data that explicitly linked indicators to flows would improve model performance. Additionally, all indicators have assumptions and thresholds that can be changed, to reflect differing local conditions or addition of new or refined flow regimes, which increases model flexibility and utility.

## Discussion

### Ecological response model outcomes and important drivers

The integrated ERM for the urban Poudre River demonstrated how the structure and function of the coupled aquatic and riparian ecosystem are strongly shaped by flow and illuminated complex interactions between different taxa and trade‐offs with different flow regimes. Thus, this model could provide restoration ecologists and managers with a tool to assess effects of potential future flows to target specific, desired processes or ecosystem attributes. Assuming additional changes from new development or climate change will cause further alterations to the urban Poudre River, the ERM would also allow insights into what specific flow components may need to be “designed” as part of any new infrastructure to help sustain or improve ecological integrity.

Our modeling led to three main observations. First, the conceptual hydrologic calendar and ERM predictions increased our understanding of the complex interactions among flows, bed mobilization, channel structure, and biota (e.g., Fig. 4) that contribute to overall ecosystem condition. Second, specific peak flow magnitudes based on geomorphic measurements and hydraulic modeling were critical for substrate cleansing and mobilization, channel morphology, and overbank flows, with strong subsequent effects on riparian and instream biota. Instream biological indicator scores (aquatic insects, native fish, trout) increased in hydrologic scenarios with greater peak flow magnitudes because of improved channel structure, the physical habitat template of the river, even though those indicators were only interactively linked to peak flows. Implicit is that other important ecological processes and communities not modeled by the ERM, including those supported by ascending or descending limb flows, are maintained. Third, an unexpected model result was that designed flows with high peaks resulted in restoration of impaired processes using about the same Poudre River annual water volume available in the flow‐depleted recent past scenario. These complex and interacting Poudre River insights demonstrated by the ERM would not be possible with more traditional flow assessments that evaluate only single variables independent of each other (Brewer et al. 2016, McManamay et al. 2016).

Modeling ERM flow effects indicated how river management could be optimized. For example, high flows had the greatest effects in the least confined channel reach, but all reaches may benefit if flow effects were combined with levee or bank modifications. To this point, lowered banks in the downstream portion of the confined reach promoted successful floodplain cottonwood recruitment in recent higher flow years. Stable base flows most effectively increased instream biological indicators such as trout and aquatic insect scores compared to present conditions because periods of stream desiccation and extreme fluctuations were reduced. Indicator scores in low peak flow scenarios were only about 50% of those with high peaks, demonstrating strong links between geomorphic function and biota.

The importance of natural flow regime components (Poff et al. 1997, Postel and Richter 2003) to a higher‐functioning Poudre River ecosystem was illustrated by ERM modeling because peak flows scoured riverbed substrate, increased channel complexity, removed excess algae, and promoted a diverse aquatic insect community that supported fish and likely, other ecosystem components such as terrestrial insectivores (e.g., Baxter et al. 2005). Extreme peak flows that may cause channel incision may not be an issue here because discharge magnitudes in designed flows are relatively low. High flows may also increase the quantity of large wood via channel migration (Yarnell et al. 2010, Wohl et al. 2017, 2016), and river connectivity to floodplain wetlands important to backwater‐dependent aquatic organisms. Descending limb flows, although not modeled explicitly, likely modified channel morphology, cued reproduction by fishes and other aquatic organisms, and prepared surfaces needed for native seed germination and seedling growth and survival necessary for perpetuating the ecologically important riparian gallery forest (Mahoney and Rood 1998, Yarnell et al. 2010). Base flows supported fish and aquatic insect reproduction and growth, and successful reproduction by trout until the spring hydrologic cycle begins again.

### A changing ecosystem

The Poudre River supports functioning remnants of native riparian and aquatic biota, but this urbanizing ecosystem has undergone significant change over the last 150 yr. Examples include channel modification and simplification, diminished native fish populations, and limited recruitment of young trees in stands of senescent narrowleaf and plains cottonwood. Native fish only approached the highest indicator condition once (stable base–high peak in the least confined reach 7) because local extinctions are exacerbated by negative modeled interactions with trout (e.g., predation) and habitat changes (e.g., backwater loss) related to simplified channel structure and, presumably, greater upstream river fragmentation and dewatering by diversion dams. Regardless, and specific to the Poudre River system, dynamic model responses of indicators demonstrated ecosystem decline was not inevitable, and that designed flows using existing and proposed infrastructure could lead to improved conditions. The flexible ERM could model ecosystem responses to additional designed Poudre River flow regimes, or be used as a general assessment approach in other altered systems where managers seek to improve ecosystem conditions, after tailoring geographically relevant indicator information for the model.

Similar to other modified arid‐land rivers, the Poudre River ecosystem is a spatially variable patchwork of physical conditions with a changing biological composition whose functioning varied even across the relatively short reaches we evaluated. For example, modeling showed confined reaches had reduced ecosystem complexity and indicator scores compared to the least confined downstream reach, which more typified pre‐development conditions (Fig. 2). Thus, modeled ecosystem responses to flow management varied in a spatial context and may better allow practitioners to align restoration prescriptions with reaches most suited for a particular management action. Extreme low flows presently occur in some Poudre River reaches and result in persistent riverbed desiccation especially in winter, effects that are exacerbated by diversion dams that limit upstream recolonization by downstream biota. Effects of management strategies to enhance river connectivity or bank restoration could be modeled in the ERM to evaluate indicator responses and relative costs and benefits of such actions.

We acknowledge that flows discussed here may benefit some nonnative species. For example, anglers fish for nonnative brown trout, because native cutthroat trout (*Oncorhynchus clarkii* [Richardson]) disappeared decades ago due to competition and hybridization with nonnative trout species (Behnke 1992, Bestgen et al. 2019). Further, predaceous trout may have a negative impact on non‐salmonid native fishes, creating a challenge in managing for healthy populations of both. We speculate that flows to benefit nonnative trout would also likely benefit native cutthroat trout that once existed here but flow management would do little to restore native trout because they were extirpated by other mechanisms (Behnke 1992).

Unlike the situation with trout, designed flows, and increased channel and floodplain management, may promote native cottonwoods via increased seedling recruitment (Merritt and Poff 2010). This is important because of limited recruitment of young trees to replace old stands of native cottonwoods, keystone species in western stream ecosystems (Merritt and Bateman 2012) that are being replaced by nonnative taxa. Thus, species‐specific responses to flow management and the relative ability to favor native taxa over nonnative ones is a planning consideration, and can be modeled with the ERM.

### Strengths and limitations of the Ecosystem Response Model

The ERM was constructed to evaluate linked biophysical responses over a range of possible flow futures, with few constraints on what is likely, affordable, or administratively possible. Decision‐makers must ultimately weigh stakeholder interests with the ecological, economic, and societal consequences associated with various policy options. Although ERM predictions are not precise in an absolute sense, the power of this modeling approach lies in its integrative and comparative nature. For example, modeling showed that instream biological indicators (e.g., algae, aquatic insects) benefitted from higher and more stable base flows and high peak flows, but stable base flows with low peak flows were only half as effective to increase indicator scores. A nuance was that trout scores in high peak designed scenarios nearly doubled when base flows changed from low to higher levels, reflecting the important seasonal role of flow on reproductive success. Thus, explicit baseflow management to enhance trout in the absence of peak flows would result in only a modest improvement in scores and at the expense of other indicators dependent on high peak flows.

Modeling also showed the strong positive link between channel structure and riparian indicators with peak flow, reflecting gradient (channel structure) or threshold (riparian) effects as peaks declined from historical flow levels. The ERM provides insight into what magnitudes of designed flows would be minimally sufficient to reestablish higher functioning along the river corridor. Thus, designed flows with high peaks would likely enhance channel and riparian functioning, but if peaks came at the expense of higher and more stable base flows, instream biota indicators would decline, demonstrating the utility of the ERM to evaluate flow scenario trade‐offs and to explore nuances that may vary seasonally or spatially.

The interactive and data‐driven ERM differs from another flow modeling approach, ELOHA, in several ways. ELOHA is mainly a multisite comparative approach intended for use in situations that are data sparse and where scientific capacity to generate detailed knowledge is lacking. Studies more detailed than ELOHA‐type analyses are required for highly valued local ecosystems, where the assumption that streamflow alone drives ecological function cannot be accepted, and where other environmental factors such as water temperature, channel structure, and streambed scour and movement, are important. The ERM for the Poudre River is such a detailed, site‐specific model that includes many relationships that are both directly and interactively influenced by flow, directly via flow‐linked pathways to indicators, and interactively through indicators. Differences notwithstanding, ERM findings could be placed into an ELOHA‐type framework by classifying the Poudre River as a particular flow regime type (in a given geomorphic context) to set expectations for the ecological performance of similar river types.

Indicator response comparisons across a set of diverse and plausible hydrologic scenarios reveal certain futures are likely better than others in terms of a highly functioning ecosystem that provides valued river amenities. Given the altered condition of the present‐day Poudre River ecosystem, managers and the public need to consider the vulnerability of the system to further hydrologic alteration and the associated trade‐offs. The ERM also illustrates another salient point for river managers to consider: that the same volume of flow can achieve substantially different ecological outcomes, depending on how it is managed.

Thus, the ERM provides a clear framework and useful decision support tool for understanding trade‐offs and consequences of various management options on water supply and biota. Indeed, a general, risk‐based modeling approach may be more useful than traditional environmental assessments that produce unintegrated measures of resource alteration, especially considering the trajectory of ecosystems under changing environmental conditions including climate warming (Schindler and Hilborn 2015). Application of probabilistic models to other systems will require the system‐specific quantification of geomorphic and ecological relationships, which will inform a transparent and science‐based process to aid decision‐making and clarify the likely trade‐offs and consequences of flow management regimes. Modeling approaches that predict ecosystem pathways also allow decision‐makers to compare a variety of stakeholder interests and the engineering, ecological, economic, and societal consequences associated with policy options (see Baker et al. 2004).

### Futures for flow‐altered systems

The ERM analyses confirmed changes in historical Poudre River ecological conditions and indicated additional legacy shifts will occur even if present flow management practices are maintained. Further, ecological changes will be accelerated by additional water development or a drying climate. However, results also indicated carefully managed flows that link key hydro‐geomorphic processes with biological responses are likely to enhance ecological functioning of the river ecosystem. Key elements of a designed flow in this and other systems similar to the Poudre River would be peak magnitudes in spring and early summer that meet threshold levels for channel maintenance and riparian vegetation, gradually ascending and descending limb flows, and relatively stable and adequate magnitude base flows, which collectively should improve geomorphic and biological indicators. Because flow requirements differ among biota, maintenance of interannual variability is important to support a more biodiverse ecosystem through time. Although we evaluated only a few designed scenarios, other flow regimes that incorporate additional seasonal or interannual variability in peak or base flows could easily be modeled to better understand those effects.

In any plausible future, the Poudre River will not return to native flows, because annual discharge in the reconstructed native scenario is up to 14× higher than other scenarios. This large gap between natural flow conditions that set the original physical template for the Poudre River and current or future flows suggests that (1) managers of heavily altered river systems may need to set ecological objectives that are not strictly “natural,” and (2) designed flows are needed to achieve specific objectives (e.g., Acreman et al. 2014, Brewer et al. 2016, McManamay et al. 2016). The ERM demonstrated that specific Poudre River objectives could be achieved with about one‐half the annual discharge of the reconstructed native scenario, if certain flow targets are met. Social and ecological benefits from designed flows in altered systems are most likely to occur if basin‐wide flow management is combined with other actions to promote upstream–downstream and channel–floodplain connectivity along the river corridor.

Additional future depletions of Poudre River flows are possible given an existing proposal to store water in a new off‐channel reservoir, which will further diminish already reduced peak flow magnitudes and impact river resources. Proposed project mitigation (Northern Colorado Water Conservancy District 2017) has focused on stabilizing base flow, which is needed to reduce present streambed desiccation. Our modeling indicated water levels to accomplish base flow functions in the stable base–high peak scenario was about 1 m^3^/s flow (about 35 cubic feet per second), the required level for successful trout reproduction (Bartholow 2010, Appendix S1: Table S2), and improved functioning of other indicators. However, the proposed base flow would meet this threshold on average only 50% of years and would not benefit river resources downstream of the city because flows will be diverted.

Peak flow frequencies and magnitudes proposed are also inadequate to maintain channel condition and biota because a 3‐d peak bypass flow is projected to occur in only 43% of years (Northern Colorado Water Conservancy District 2017; data *available online*).^1^ Further, mean peak Poudre River flow magnitudes are unlikely to reach even the 31 m^3^/s estimated for the relatively low present operations scenario in most years. As modeled by the ERM and predicted by fundamental principles of river science (Poff et al. 1997, Wohl et al. 2015), changes from proposed additional water development would essentially ensure a general and long‐term decline in Poudre River aquatic and riparian ecosystem functions. Thus, the best possibility for maintaining or improving Poudre River ecological conditions with the proposed off‐channel storage is designed peak flows that bypass the newly proposed storage reservoir for a minimum of three consecutive days with the predicted highest magnitude flows each year. This scenario also ensures the natural interannual variability in flows needed to sustain ecosystem functioning, effects of which are seen by comparing ERM outcomes of managed scenarios with different peak flow levels.

Ideally, the frequency and magnitude of peak flows in flow‐depleted rivers could be partially restored to more closely approximate natural flows, which here are those in the reconstructed native scenario (i.e., ≥3‐d peak flows in more than 50% of years that reach 94.9 m^3^/s at Fort Collins, to provide the flow magnitude and duration needed for channel maintenance (Andrews and Nankervis 1995, Emmett and Wolman 2001)). Although existing storage reservoirs and diversions have substantially reduced Poudre River peak flows, our analyses show that the estimated “deficit” in peak flow volume and duration could be met with bypasses from existing storage facilities or diversions in the Poudre River basin, which in real time would require adequate flow forecasting. Other studies that have implemented designed flows (Kiernan et al. 2012) or modeled them (Chen and Olden 2017, Sabo et al. 2017) show it is feasible to balance existing human demands while provisioning key ecosystem targets. Adaptive management will be needed to ensure flow scenarios support desired outcomes. Additional details regarding the high flow mitigation specific to the Poudre River are elsewhere (Appendix S2).

As stressors on over‐allocated river ecosystems increase from human water demands and climate change, modeling approaches that predict future ecosystem responses to water development and management will play an increasingly important role in informing public debate and choices about management of these resources (Baker et al. 2004, California State Water Resources Control Board. 2017). Ecosystem‐based models such as the ERM can identify strategies to achieve firm targets to assist with rehabilitation or mitigation plans in water development scenarios. Unfortunately, no policy requires that integrated, holistic, ecosystem‐scale impacts be assessed before new water projects are approved. Rather, requirements for assessing “impact” under NEPA are satisfied when analyses are framed only in traditional single‐variable models. Thus, even when river engineers and other scientists not associated with water development interests construct holistic models of “impact” (e.g., the ERM), there is no clear pathway to having those substantively considered in project development, much less adopted. Another fundamental problem with the traditional NEPA‐driven “environmental impact” approach is failure to consider ecosystem functions and societal values on par with the economic factors that largely dictate proposed alternatives for development. Typically, impacts of the preferred project alternative are evaluated with a few single‐factor analyses that are portrayed as causing minimal environmental alteration. Joint consideration of both long‐term ecological issues and short‐term economic gain at the project proposal stage may aid development of more environmentally sustainable alternatives, especially in light of new uncertainties posed by climate change (see Poff et al. 2016). This would promote more robust science and more transparent trade‐off analyses of alternative development options needed to support more rational societal decisions about river management in a complex and uncertain future.

## Data Availability

Data are available from the City of Fort Collins Natural Areas Department at https://www.fcgov.com/naturalareas/pdf/erm_report.pdf, https://www.fcgov.com/naturalareas/pdf/erm_appendix.pdf?1421099850, and https://www.fcgov.com/naturalareas/eco-response.php

## Note

1 http://www.northernwater.org/docs/NISP/MapsDocuments/2017FWMEPFinal.pdf
