# Supplementary material for: Designing flows to enhance ecosystem functioning in heavily altered rivers
Source: Ecol Appl. 2019 Oct 18;30(1):e02005. doi: 10.1002/eap.2005 (PMC9285520; doi:10.1002/eap.2005)
Supplement: Supplementary file 1 [file EAP-30-e02005-s003.pdf]

Supporting Information. Bestgen, K. R., N. L. Poff, D. W. Baker, B. P. Bledsoe, D. M. Merritt, M. Lorie, G. T. Auble, J. S. Sanderson, and B. C. Kondratieff. 2019. Designing flows to enhance ecosystem functioning in heavily altered rivers. *Ecological Applications*.

Appendix S1

CONTENTS

Table S1: Flow statistics of the Poudre River.

Table S2: Hydrologic metric summary for Poudre River flow scenarios.

Table S3: Summary of indicator-specific tasks and data inputs for each indicator variable.

Table S4: Historical, Future, and Designed flow scenarios modeled with the Poudre River Ecological Response Model.

Appendix S1: Table S1. Flow statistics for reaches of the Poudre River moving in an upstream to downstream direction.† Reach 3a is the confined reach, 3b the moderately confined reach, and 7 is least confined.

	Canyon Gage	Below Greeley Diversion	Below Hansen Canal	Below Pleasant Valley Pipeline	Below Jackson Ditch	Below New Mercer Diversion	Below Arthur Ditch	Below Larimer and Weld Diversion	Below Lake Canal	Lincoln Gage	Boxelder Ditch	Boxelder Gage
ERM reaches						1	2	3a+3b	4	5	6	7
Median (m^3/sec)	1.90	1.42	1.98	1.81	1.64	1.27	1.39	0.88	0.82	0.85	1.02	0.37
Mean (m^3/sec)	9.17	8.72	11.41	10.93	8.38	7.28	7.33	4.64	4.33	4.36	4.47	3.77
Standard deviation (mean)	16.45	16.48	17.78	17.36	14.95	14.02	13.99	12.18	11.98	12.03	11.89	11.55
Coefficient of variation (%)	5.1	5.4	4.5	4.5	5.1	5.4	5.4	7.4	7.9	7.9	7.6	8.8
Number of zero flow days	0	449	0	164	29	884	5	1848	70	27	66	0
Percent zero flow days	0.0%	3.0%	0.0%	1.1%	0.2%	5.9%	0.0%	12.3%	0.5%	0.2%	0.4%	0.0%

† Based upon a Recent Past scenario, which is a daily point flow model using 40 years (WY 1970-2010) of historical gage data and operations records as developed by the Northern Colorado Water Conservancy District.

Appendix S1: Table S2. Hydrologic metric summary for Historical, Future, and Designed flow scenarios for the Poudre River downstream of Larimer and Weld Canal.

	Historical Hydrologic Scenarios			Future Scenarios		Designed Hydrologic Scenarios			
Hydrologic Metric	Reconstructed Native	Recent Past	Present Operations	Additional Water Development	Driest Climate	StableBase-LowPeak	HighBase-ModeratePeak	DryBase-HighPeak	StableBase-HighPeak
Period of record (Water Year)	1950–2005	1970–2010	1950–2005	1950–2005	1950–2005	1950–2005	1950–2005	1950–2005	1950–2005
Average daily flow (m^3/sec)	10.85	4.64	2.80	1.36	0.76	1.84	5.15	3.77	4.16
Average summer (July–September) base flow (m^3/sec)	9.83	3.57	2.29	1.47	0.37	1.67	5.32	1.90	1.73
Average winter base flow (m^3/sec)	1.98	0.68	0.34	0.14	0.11	0.68	1.56	0.59	0.99
Average peak daily flow (m^3/sec)	93.1	55.1	31.0	14.8	13.4	28.3	53.8	72.3	71.8
Wetland flow (m^3/sec)†	74.9	46.4	28.5	12.0	7.0	19.8	28.3	63.7	63.7
Functioning riparian zone flow (m^3/sec)‡	118.1	108.5	62.0	42.1	22.5	30.6	49.6	94.9	94.9
5-year return period flow (m^3/sec)	119.2	79.1	44.8	14.8	16.3	34.0	58.0	94.9	94.9
Percent zero flow days	0.0%	12.3%	30.7%	40.0%	30.7%	0.0%	0.0%	30.4%	0.0%
Annual volumetric discharge (ha-m)	34,246	14,640	8,881	4,383	2,382	5,785	16,259	11,908	13,117

† Flow occurring two weeks of the growing season, May–September, every other year (later described in more detail in the Riparian Vegetation section; Shanahan et al. 2014).

‡ Flow occurring one day per year (described in more detail in the Riparian Vegetation section, Shanahan et al. 2014).

Appendix S1: Table S3. Summary of indicator-specific tasks and data inputs for each indicator variable. The ERM input flow scenarios include magnitude, duration, and frequency of flows, and for biota, base flow levels. Poudre River geometry is from a HEC RAS model developed by the City of Fort Collins (City) for FEMA flood mapping. Reach 3a = confined reach, 3b = moderately confined reach, and 7 = least confined reach. Additional descriptions in Shanahan et al. (2014).

Indicator	Relevance	Data Inputs	Analysis Summary
Channel structure	Combined influence of substrate conditions, channel geometry as physical template for physical and ecological processes	• Comprehensive evaluation of available grain size<br>• Poudre River geometry<br>• Historical aerial images<br>• ERM flow scenarios	Evidence of thresholds for sediment transport based on analyses of (1) dimensionless shear stress, (2) effective discharge, and (3) historical aerial images. Channel maintenance flow index accounts for the influence of flow magnitude, duration, and frequency on sediment mobilization and channel migration.
Algae	Basal aquatic food web resource, but excessive nutrients create unaesthetic growths	• City water quality data, 1980-2005<br>• Process-based relationships between nutrient enrichment and algae growth<br>• ERM flow scenarios	Assemble daily single nutrient measurements into average annual loads as a function of daily average discharge. Exceedance probabilities over threshold concentrations for nuisance algae growth were used to calculate enrichment.
Aquatic insects	Abundance and species distribution of organisms, as indicators of water quality and food web structure	• Benthic macroinvertebrate data from literature and Colorado State University (CSU)<br>• ERM flow scenarios	% of all species in orders Ephemeroptera (mayflies), Plecoptera (stoneflies), and Trichoptera (caddisflies) for April and August, and expert knowledge of (1) aquatic insect life cycles, (2) environmental tolerances, and (3) substrate preferences to relate to the influence of the channel structure, summer base flow/temperature, and algae.
Native fish	Species richness and abundance reflect relative health and condition of biotic ecosystem	• K. Bestgen data (1993-2014) describing native fish richness, abundance, and community biomass from reaches 3 and 7<br>• Process-based knowledge of aquatic ecosystem interactions<br>• ERM flow scenarios	Patterns of native fishes and total fish species richness were examined (number of each species at a site), fish abundance (number of all individuals collected), and fish community biomass at the McMurray Natural Area (ERM Reach 3b) and ELC (ERM Reach 7) sites relative to various flow metrics and relative to year (time effect).
Trout	Valued recreational sport fish as a component of the ecosystem and indicator of the thermal and hydrologic regime, that is especially sensitive to base flow level	• K. Bestgen data (1993-2007) of trout abundance and biomass from Reach 3<br>• Process-based knowledge of aquatic ecosystem interactions<br>• ERM flow scenarios	Brown trout winter flow thresholds based upon the relationship between average flow and number of young-of-year brown trout. Additional factors of summer base flow/temperature, channel structure, and aquatic insect abundance/diversity related using process-based knowledge of ecosystem interaction.
Rejuvenating mosaic forest	Multi-stage forest dominated by riparian species adapted to disturbance-prone environments (e.g., plains cottonwood) that dominates the river ecosystem	• Poudre River geometry<br>• ERM flow scenarios	Calculated as product of (1) area (expressed as average width) of potential floodplain turnover (low-lying, occasionally flooded area where movement is most likely), (2) ability of flow to mobilize substrate based on shear stress, and (3) a reduction due to extent of bank stabilization. Inundation surfaces developed using HEC RAS model.

Indicator	Relevance	Data Inputs	Analysis Summary
Functional riparian zone	Riparian zone hydrologically connected to the river and may support biogeochemical processing, flood peak attenuation, sediment trapping and exchange, episodic expansion of aquatic habitat, and productive vegetative communities	• City riparian vegetation data<br>• Poudre River geometry<br>• ERM flow scenarios	Inundation surfaces developed using HEC RAS model. Represents area inundated by river at least one day in two years of growing season days. Vegetation transect data confirms substantial differences in plant species composition above (drier) compared to below (wetter) the stage of the 0.0033 exceedance flow.
Riparian wetland width	Area where river inundation sufficient to support plant communities dominated by wetland species	• City riparian vegetation classification<br>• Poudre River geometry<br>• ERM flow scenarios	Inundation surfaces from HEC RAS model. Wetlands are land inundated 5% of all growing season (May–September) days. Corresponds to flow exceedance values associated with a 50% probability of occurrence of the hydrophytic vegetation.

Appendix S1: Table S4. Historical, Future, and Designed flow scenarios modeled with the Poudre River Ecological Response Model (ERM). Flow rates and annual water volumes required for scenarios presented in Table S2. City = City of Fort Collins.

Name & purpose	Period of record*	General description	Source
Historical scenarios			
Reconstructed Native	1949-2005	Flows for the Poudre River with human influences removed; native flow conditions.	Monthly† model that factors in water diversions, trans-basin augmentation (water added to the Poudre from other watersheds), return flows, and retimes the water held back in reservoirs to the historical hydrologic record. Developed in a MODSIM‡ model by the City.
Recent Past	1969-2010	Historical gaged flow data, includes historical climate variability and operations§ over time.	Daily point flow model based on historical gage data (Canyon Mouth, Lincoln Street, and Boxelder) and operation records of diversion structures as developed by the Northern Colorado Water Conservancy District (NCWCD).
Present Operations	1949-2005	River flows with current operations imposed on period of record; different from Recent Past scenario because more water is diverted than in the 1950s.	Monthly† model that applies 2010 operations to the Reconstructed Native scenario. Developed in MODSIM model by City.
Future scenarios			

Additional Water Development	1949-2005	Includes present conditions and estimated flow alteration from construction and operation of three proposed water projects.	Monthly† model that applies projected 2050 operations to the Reconstructed Native scenario. Developed in a MODSIM model by City. Data and models used were preliminary, and may differ from the final hydrology determined in EIS.
Driest Climate	1949-2005	Includes present conditions and superimposes driest conditions forecast from climate models.	Present Operations scenario data modified using driest climate projections and bias corrected and downscaled WCRP CMIP3 Climate and Hydrology Projections.
Designed scenarios			
StableBase-LowPeak	NA	Similar to Additional Water Development scenario except base flows consistent.	Shanahan et al. 2014
HighBase-ModeratePeak	NA	Uses 25th percentile of monthly average native flows and recommended managed changes in monthly streamflow, including a moderate peak magnitude.	Bartholow 2010

DryBase-HighPeak	NA	Ample peak flows, low/no and variable base flows, % dry days patterned on Present Operations scenario and high flows on StableBase-HighPeak scenario.	Shanahan et al. 2014
StableBase-HighPeak	NA	Flow regime designed with minimum flow needed to elevate ERM indicators to highest levels.	"

* All years are water year from Nov 1 to Oct 31
† Later disaggregated to daily using historical data from the USGS Poudre Canyon Mouth gage:
[online] URL: http://www.dwr.state.co.us/SurfaceWater/data/detail_graph.aspx?ID=CLAFTCCO.
‡ [online] URL: http://modsim.engr.colostate.edu/
§ *Operations* refers to all trans-basin basin diversions, diversion withdrawals, and reservoir storage.
[online] URL: http://gdo-dcp.ucllnl.org/downscaled_cmip_projections/dcpInterface.html.

Literature Cited

Bartholow, J. M. 2010. Constructing an interdisciplinary flow regime recommendation. Journal of the American Water Resources Association 46(5):892–906.

Shanahan J. O., D. W. Baker, B. P. Bledsoe, N. L. Poff, D. M. Merritt, K. R. Bestgen, G. T. Auble, B. C. Kondratieff, J. G. Stokes, M. Lorie and J. S. Sanderson. 2014. An Ecological Response Model for the Cache la Poudre River through Fort Collins. City of Fort Collins Natural Areas Department, Fort Collins, CO. 93 pp + appendices.
