# Supplementary material for: Designing flows to enhance ecosystem functioning in heavily altered rivers
Source: Ecol Appl. 2019 Oct 18;30(1):e02005. doi: 10.1002/eap.2005 (PMC9285520; doi:10.1002/eap.2005)
Supplement: Supplementary file 2 [file EAP-30-e02005-s002.pdf]

Supporting Information. Bestgen, K. R., N. L. Poff, D. W. Baker, B. P. Bledsoe, D. M. Merritt, M. Lorie, G. T. Auble, J. S. Sanderson, and B. C. Kondratieff. 2019. Designing flows to enhance ecosystem functioning in heavily altered rivers. *Ecological Applications*.

Appendix S2

CONTENTS

Enhanced description of consequences of future Poudre River water depletions and recommended flows.

Additional future depletions of Poudre River flows are possible given an existing proposal to divert and store water in a new off-channel reservoir. The proposed Northern Integrated Supply Project (NISP; U. S. Army Corps of Engineers 2018) must comply with the 1970 National Environmental Policy Act (NEPA) and the 1972 Clean Water Act, and thus, has developed an environmental impact analysis and mitigation plan. Proposed storage will further diminish already reduced peak flow magnitudes and inevitably affect channel condition and in-channel and riparian biota. Proposed project mitigation (Northern Colorado Water Conservancy District 2017) has focused on stabilizing base flow, which is needed to reduce seasonally desiccated areas and increase ecosystem functioning during those periods. Our analyses indicate water levels to accomplish base flow functions in the Stable Base-High Peak scenario are relatively low, and consistent with empirical relationships that indicated about 1 m^3/sec flow (about 35 ft^3/sec) was required for successful brown trout reproduction (Bartholow 2010; Appendix S1; Table S2). Yet, the base flow proposed by NISP would meet this threshold on average only 50% of year. In addition, proposed improvements to base flow conditions in the urban corridor will not be realized throughout the river because base flows will be diverted just downstream of the City and stored in reservoirs.

Peak flow frequencies and magnitudes proposed in the mitigation plan (Northern Colorado Water Conservancy District 2017) are inadequate to maintain channel condition, and instream and riparian biota. For example, the only condition under which a 3-day peak bypass flow would occur is if the reservoir is filled to ≥ 76% of capacity and is projected to fill that year, a condition predicted in only 43% of years (http://www.northernwater.org/docs/NISP/MapsDocuments/2017FWMEPFinal.pdf; Northern Colorado Water Conservancy District 2017). Therefore, in nearly 60% of future years, the largest

peak flow proposed is only 79.3 m^3/sec, and for a maximum of only 2 days, a level that will not meet the thresholds necessary to maintain critical river ecosystem functions. Additionally, that flow magnitude is not likely to be achieved in our more downstream study area because the mitigation plan proposes to provide those flows only at the project's point of diversion upstream from the City. In addition to proposed project diversions up to 28.3 m^3/sec, several large water diversions in that intervening section can reduce normal peak flow volumes by an additional 50% or more (Appendix S1; Table S1). Thus, mean peak Poudre River flow magnitudes are unlikely to reach even the 31 m^3/sec estimated for the relatively low Present Operations scenario in most years. Proposed flows are also problematic because peak magnitudes required to maintain channel conditions function essentially in a non-linear manner, such that thresholds for incipient motion of the streambed must be attained to be effective. In other words, proposed reductions of peak flows will result in disproportionately less channel work and sediment transport capacity and increased channel simplification due to vegetation encroachment. Reduced sediment transport and channel narrowing will likely also cause increased flooding risk during extreme events due to increased flow resistance and bed aggradation. As modeled by the ERM and predicted by fundamental principles of river science (Poff et al. 1997, Wohl et al. 2015), changes from proposed additional water development would essentially ensure a general and long-term decline in Poudre River aquatic and riparian ecosystem functions.

A Poudre River flow regime modeled after the naturally-patterned Stable Base-High Peak scenario resulted in substantially increased Channel structure and associated ecological indicator scores (Figure 5). Thus, our main recommendation to maintain or improve Poudre River ecological conditions is designed peak flows that bypass the newly proposed storage reservoir for a minimum of three consecutive highest magnitude days each year. This will ensure that in

each year a “natural” peak runoff will occur that can exert work on the riverbed over a range of high flows, including occasional years when those flows exceed critical thresholds. This scenario also ensures the natural variability in flows needed to sustain ecosystem functioning.

Ideally, the frequency and magnitude of peak flows could be restored to approximate those in the Reconstructed Native scenario, i.e., ≥ 3-d peak flows in more than 50% of years that reach 94.9 m^3/sec at Fort Collins (USGS gage 06752260). Such a regime could provide the flow magnitude and duration consistent with long-term channel maintenance for regional rivers (Andrews and Nankervis 1995; Emmett and Wolman 2001). However, existing storage reservoirs and diversions in the Poudre River watershed have substantially reduced peak flows; the 42-yr hydrologic record (1976-2017, gage 06752260) showed flows reached those levels in only nine years (21% frequency; includes a September 2013 flood event and two years [1979 and 1995] that were slightly [2 m^3/sec] below those levels). Calculations showed achieving those high magnitude peaks in 12 additional years (21 of 42 total) required an average of 31 m^3/sec (range 11.6-61.0 m^3/sec) additional water on each of the three peak flow days each year. Further, our analyses show that the estimated “deficit” in peak flow volume and duration could be met with bypasses from existing storage facilities or diversions in the Poudre River basin, which in real time would require adequate flow forecasting.

Therefore, we propose as an additional, longer-term recommendation, investigating the feasibility of providing coordinated bypasses at diversions and storage facilities (both proposed and existing) in the Poudre River basin to meet the peak flow magnitudes and durations necessary to fully support the Poudre River ecosystem. In Colorado, mechanisms to support such a recommendation can be facilitated through the Colorado Water Trust (https://coloradowatertrust.org/). A designed flow scenario with bypasses as described shares the

burden of improved ecological conditions in the Poudre River among historical and new users and provides a starting point for discussions among basin water managers for holistic river management. Other studies that have implemented designed flows (Kiernan et al. 2014) or modeled them (Chen and Olden 2017, Sabo et al. 2017) have shown it is feasible to balance existing human demands while provisioning key ecosystem targets. Adaptive management will be needed to ensure flow scenarios support desired outcomes.

Literature Cited

Andrews, E. D., and J. M. Nankervis. 1995. Effective discharge and the design of channel maintenance flows for gravel-bed rivers. Pages 151-164, *in* J. E. Costa, A. J. Miller, K. W. Potter, and P. R. Wilcock (eds). Natural and Anthropogenic Influences in Fluvial Geomorphology, American Geophysical Union, Geophysical Monograph 89.

Chen, W., and J. D. Olden. 2017. Designing flows to resolve human and environmental water needs in a dam-regulated river. Nature Communications 8, Article number: 2158. doi:10.1038/s41467-017-02226-40

Emmett, W. W., and M. G. Wolman. 2001. Effective discharge and gravel-bed rivers. Earth Surface Processes and Landforms 26:1369-1380.

Kiernan, J. D., P. B. Moyle, and P. K. Crain. 2012. Restoring native fish assemblages to a regulated California stream using the natural flow regime concept. Ecological Applications 22:1472-1482.

Northern Colorado Water Conservancy District. 2017. Fish and wildlife mitigation and enhancement plan. Prepared for the Colorado Parks and Wildlife Commission, by the

Northern Colorado Water Conservancy District. Berthoud, Colorado. http://www.northernwater.org/sf/WaterProjects/NISP . http://www.northernwater.org/docs/NISP/MapsDocuments/2017FWMEPFinal.pdf

Poff, N. L., J. D. Allan, M. B. Bain, J. R. Karr, K. L. Prestegaard, B. Richter, R. Sparks, and J. Stromberg. 1997. The natural flow regime: a new paradigm for riverine conservation and restoration. BioScience 47:769–784.

Sabo, J. L., A. Ruhí, G. W. Holgrieve, V. Elliott, M. E. Arias, P. B. Ngor, T. A. Räsänen, and S. Nam. 2017. Designing river flows to improve food security futures in the Lower Mekong Basin. Science 358, 6368, eaao1053 (DOI: 10.1126/science.aao1053)

U. S. Army Corps of Engineers. 2018. Final environmental impact statement: Northern Integrated Supply Project. Omaha District, Omaha, Nebraska. http://www.nwo.usace.army.mil/Missions/Regulatory-Program/Colorado/EIS-NISP/

Wohl, E., B. P. Bledsoe, R. B. Jacobson, N. L. Poff, S. L. Rathburn, D. M. Walters, and A. C. Wilcox. 2015. The natural sediment regime: broadening the foundation for ecosystem management. BioScience 65:358–371.
