# Supplementary material for: Designing flows to enhance ecosystem functioning in heavily altered rivers
Source: Ecol Appl. 2019 Oct 18;30(1):e02005. doi: 10.1002/eap.2005 (PMC9285520; doi:10.1002/eap.2005)
Supplement: Supplementary file 3 [file EAP-30-e02005-s004.pdf]

Bestgen, K. R., N. L. Poff, D. W. Baker, B. P. Bledsoe, D. M. Merritt, M. Lorie, G. T. Auble, J. S. Sanderson, and B. C. Kondratieff. 2019. Designing flows to enhance ecosystem functioning in heavily altered rivers. *Ecological Applications*.

Data S1

Data to reproduce probability tables and final outcomes of the ecological response model (ERM), including conditional probability tables for all eight indicators of ecosystem conditions (FinalERM_Conditional_probability_tables_All.txt) and the flow and water temperature driver, and daily flow data for water years in the period 1950-2005 for nine hydrologic scenarios (Final_ERMflowscenario_Data_All.txt) in "Designing flows to enhance ecosystem functioning in heavily altered rivers". The narratives (ERMChannelStructureCaseStudyNarrative.pdf, and ERMBrownTroutCaseStudyNarrative.pdf) and data for case studies for channel structure and trout indicator variables are provided as .pdf's and the computational formulae (ERMcase studies_manuscript_ChannelStructure.csv and ERMcase studies_manuscript_BrownTrout.csv) are provided as .csv files. Examples, including linked spreadsheets, are referenced in the text (Shanahan et al. 2014; and https://www.fcgov.com/naturalareas/eco-response.php.

Authors of the material provided in DataS1.zip

Kevin R. Bestgen
Department of Fish, Wildlife and Conservation Biology and the Graduate Degree Program in Ecology, Colorado State University, 1474 Campus Delivery, Fort Collins, CO 80523, USA
kbestgen@colostate.edu

N. LeRoy Poff
Department of Biology and Graduate Degree Program in Ecology, Colorado State University, Fort Collins, CO 80523 USA, and Institute for Applied Ecology, University of Canberra, Bruce ACT 2617, Australia
N.Poff@colostate.edu

Daniel W. Baker
Colorado State University, Department of Civil and Environmental Engineering, Fort Collins, CO, 80523 USA
Dan.baker@colostate.edu

Brian P. Bledsoe^
Colorado State University, Department of Civil and Environmental Engineering, Fort Collins, CO, 80523 USA
bbledsoe@uga.edu

David M. Merritt
USDA Forest Service, National Stream and Aquatic Ecology Center, 2150 Center Ave, Fort Collins, CO 80526 USA
dmmerritt@fs.fed.us
Mark Lorie
Corona Environmental Consulting, 357 McCaslin Blvd., Louisville, CO 80027 USA
mlorie@coronaenv.com

Gregor T. Auble
U. S. Geological Survey, Fort Collins Science Center, 2150 Center Ave. Fort Collins, CO, 80526 USA
aubleg@usgs.gov

John S. Sanderson
The Nature Conservancy, 2424 Spruce St., Boulder, CO 80302 USA
jsanderson@TNC.ORG

Boris C. Kondratieff
Colorado State University, Department of Bioagricultural Sciences and Pest Management, 1177 Campus Delivery, Fort Collins, Colorado 80523 USA
Boris.Kondratieff@colostate.edu

^ Present address, University of Georgia, Athens, Georgia, 30602

File list (files found within DataS1.zip)

```
Final ERM_Conditional_probability_tables_All.txt
Final_ERMflowscenario_Data_All.txt
ERMChannelStructureCaseStudyNarrative.pdf
ERMBrownTroutCaseStudyNarrative.pdf
ERMcase studies_manuscript_ChannelStructure.csv
ERMcase studies_manuscript_BrownTrout.csv
```

Description

Final ERM_Conditional_probability_tables_All.txt – Text file for conditional probability tables for eight indicators of ecosystem conditions and the flow and water temperature driver for the ecological response model (ERM).

Final_ERMflowscenario_Data_All.txt – Text file for daily flow data for the water years in the period 1950-2005 for nine hydrologic scenarios for the ecological response model (ERM).

ERMChannelStructureCaseStudyNarrative.pdf – pdf to explain the flow of the Channel Structure ERM model and portrayal of all data and computations (expand to about 400x) and an example outcome.

ERMBrownTroutCaseStudyNarrative.pdf – pdf to explain the flow of the Brown Trout ERM model and portrayal of all data and computations (expand to about 500x) and an example outcome.

ERMcase studies_manuscript_ChannelStructure.csv – .csv file to recreate formulae to derive Channel Structure model output.

ERMcase studies_manuscript_BrownTrout.csv – .csv file to recreate formulae to derive Brown Trout model output.
