# Supplementary material for: Designing flows to enhance ecosystem functioning in heavily altered rivers
Source: Ecol Appl. 2019 Oct 18;30(1):e02005. doi: 10.1002/eap.2005 (PMC9285520; doi:10.1002/eap.2005)
Supplement: Supplementary file 4 [file EAP-30-e02005-s001.zip › ERMBrownTroutCaseStudyNarrative.pdf]

ERM Trout Case Study - Hydrology: Recent Past, Reach: 3a

Case Study Overview

To navigate this case study navigate the worksheet from left to right. Click on any cells as all the computational linkages have been maintained.

1. Conceptual Diagram - road map of the ERM computations and the information on this page.
2. Narration - Details the analysis performed in each step.
3. Pre-Model Probability Computations - performed outside Genie Model
- **3a. Single-Variable Probability Tables + Weights**
- **3b. Partial Probability Table for Each State**
- **3c. Pre-Model Probability Table**

4. Pre-Model Probabilities from other contributing variables - performed
5. Model Computations
5. Final Results

1. Conceptual Diagram

A - Fundamental Probability Relationships

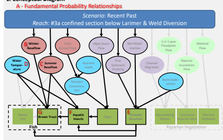

2. Narration

In this case study of the numerical computation of the model for the prediction of Trout condition, the nodes in bold and with red call-out letters (from Figure 1. Conceptual Diagram to the left) will be discussed. Trout computations are outlined in the following steps:

A. The probability model (Genie) required fundamental relationships between all combinations of the four contributing variables and Brown Trout condition. The steps to develop these fundamental relationships started with a process to relate the single-variables of winter baseflow, summer baseflow/temperature, channel structure, and aquatic insects to the four possible trout states (see Table 3a on this worksheet). Additionally, weights were applied both across variables and within the states of each variable. The within-state weighting was done to factor in limiting factors (e.g. channel structure is more influential if it is in good condition (55% weight for 'Clean and diverse') vs. a poor condition (10% weight for 'Entrenched'). Next the probabilities and weights were combined for each variable (Table 3b on this worksheet) and finally collected across variables (Table 3c on this worksheet). The probabilities from the 3c. Pre-Model Probability Tables were then uploaded into the probabilistic Genie model to evaluate the impact of the various flow scenarios on Trout.

B. The input variable of Winter Baseflow conditions was computed from the daily record of the Recent Past Scenario. **For Reach 3a it was found that 75-90% of years had an average winter baseflow less than the critical level of 35 cfs** for overwinter recruitment.

C. The input variable of Summer Baseflow conditions was also computed from the daily record for the Recent Past Scenario. **For Reach 3a there was a 100% probability that summer base flow levels were lower than the minimum threshold** (defined as an average flow greater than 35 cfs and daily minimum flow above 20 cfs)

D. The input variable of Temperature is recognized as a key driver for Trout, however little historical water temperature data was available. Therefore, summer **water temperature was set to a 50/50 probability of being above or below 20°C**.

E. The input variable of Channel Structure (which was computed in the previous page of this worksheet) is described **by a distribution of there being a 26% probability of the Channel Structure being Entrenched, 50% largely immobile and homogeneous, 22% Partially mobile and diverse, and 2% Clean and diverse**.

F. The input variable of Aquatic insects **was predicted from related computations to have a 50% chance of falling into the "-" category, a 48% change of falling into the "0" category, and a 2% chance of falling into the "+" category.**

G. The probabilistic Genie model then combined the data from B-F above (as listed in Table 4 Pre-Model Probabilities from Other Variables on this worksheet) with the data from the 3c. Pre-Model Probability Table to perform the Model Computations (reproduced as Table 5 in this worksheet). Thus for the give inputs, **Trout are thus predicted to have a 42% chance of falling into the "- -" category, 30% chance of falling into the "-" category, a 23% change of falling into the "0" category, and a 5% chance of falling into the "+" category.** Finally, a single expected value was computed to represent the final probability distribution.

3a. Single-Variable Probability Tables + Weights

Based upon fundamental relationships

Brown Trout States

Winter baseflow table — Table Weight 0.2709

% of years <35 cfs	--	-	0	+	State Weights: by State	State Weights: Net
0% to 25%	0	0.1	0.2	0.7	0.1	0.02709
25% to 50%	0.1	0.2	0.3	0.4	0.15	0.040635
50% to 75%	0.2	0.3	0.3	0.2	0.2	0.05418
75% to 90%	0.5	0.4	0.1	0	0.25	0.067725
>90%	0.9	0.1	0	0	0.3	0.08127
Sum					1	0.2709

Summer Baseflow + Temp — Table Weight 0.2708

Qavg > 35cfs & Qmin > 20cfs, Tmax 20 C	--	-	0	+	State Weights: by State	State Weights: Net
>TsumQ & <Ttemp	0	0	0.5	0.5	0.1	0.02708
>TsumQ & >Ttemp	0.5	0.5	0	0	0.15	0.04062
<TsumQ & <Ttemp	0	0.5	0.5	0	0.2	0.05416
<TsumQ & >Ttemp	1	0	0	0	0.55	0.14894
Sum				Sum	1	0.2708

Channel Structure — Table Weight 0.2708

As a function of high flows and geometry	--	-	0	+	State Weights: by State	State Weights: Net
Clean & Diverse	0	0	0.25	0.75	0.55	0.14894
Partially Mobile and diverse	0	0.25	0.5	0.25	0.2	0.05416
Largely immobile and homogeneous	0	0.5	0.5	0	0.15	0.04062
Entrenched	0.75	0.25	0	0	0.1	0.02708
Sum					1	0.2708

Aquatic Insects — Table Weight 0.1875

As a function of water temp and baseflow	--	-	0	+	State Weights: by State	State Weights: Net
+	0	0	0.5	0.5	0.5	0.09375
0	0	0.25	0.5	0.25	0.3	0.05625
-	0.25	0.5	0.25	0	0.2	0.0375
Sum					1	0.1875
Column of table	2	3	4	5		

Table II.18: Description of four states of Brown Trout that depend on summer temperature, summer baseflow, winter baseflow, Aquatic Insects, and Channel Structure.

State	Definition
+	Multiple (3–4 or more) age classes; successful annual reproduction; high total biomass; resilient to multiple detrimental events/years; viable recreational fishery, many adult fish.
0	Three age classes; more variability across years in terms of biomass and reproduction; variable as a recreational fishery from year to year, occasional years with moderate numbers of adult fish.
-	Dominated by a single age class, others may be present; reproduction minimal; recovery from stressor events would take several years; generally poor fishery, inconsistent from to year to year.
--	Single age class present; very sporadic reproduction; low abundance, population vulnerable to one detrimental event/year, the poor fishery is in danger of collapse; many years of good conditions for recovery.

Table number (II.18) corresponds to that in Shanahan et al. 2014

3b. Partial Probability Table for Each State

((single-variable probability) * (net weight))

[illegible]

3c. Conditional Probability Table

(sum of partial probability for each state/(total weight))

Probabilities for Brown Trout

[illegible]

4. Pre-Model Probabilities from Other Variables

for the same scenario

Winter Baseflow: % years avg <20 cfs

Scenario	Reach
Historic	3a
0% to 25%	0
25% to 50%	0
50% to 75%	0
75% to 90%	1
>90%	0

Summer Baseflow and Water Temp

Scenario	Reach
Historic	3a
>TsumQ & <Ttemp	0
>TsumQ & >Ttemp	0
<TsumQ & <Ttemp	0.5
<TsumQ & >Ttemp	0.5

Channel Structure

Scenario	Reach
Historic	3a
Clean and diverse	0.021
Partially mobile and diverse	0.224
Largely immobile and homogeneous	0.497
Entrenched	0.258

Aquatic Insects

Scenario	Reach
Historic	3a
+	0.0200
0	0.4833
-	0.4967

5. Model Computations

Bringing together pre-model probabilities and the probabilities of other variables

[illegible]

5. Final Results

Final probabilities for Brown Trout state

--	-	0	+
0.4215	0.2980	0.2328	0.0477

[illegible]

Full Distribution

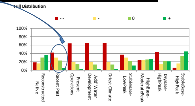

Single Expected Value, reported in Figure 6 of manuscript

State	--	-	0	+
Prob for each state	0.4215	0.2980	0.2328	0.0477
Scaling parameters	0.000	0.333	0.666	1.000
Partial probabilities	0.000	0.099	0.155	0.048
Expected Value				0.302
