# Supplementary material for: Designing flows to enhance ecosystem functioning in heavily altered rivers
Source: Ecol Appl. 2019 Oct 18;30(1):e02005. doi: 10.1002/eap.2005 (PMC9285520; doi:10.1002/eap.2005)
Supplement: Supplementary file 4 [file EAP-30-e02005-s001.zip › ERMChannelStructureCaseStudyNarrative.pdf]

ERM Channel Structure Case Study - Hydrology: Recent Past, Reach: 3a

<u>Case Study Overview</u>
To navigate this example read the information from left to right.

- Look at the **1. ERM Conceptual diagram** (below). All bold nodes contribute to channel structure. The red capital letters provide a reference to the lettered steps in the Narration.
- Read the **2. Narration** for a synthesis of the steps involved for this particular example. Note that the computational steps in the spreadsheet are also cross referenced to these lettered steps.
- Investigate the computations detailing the **3. Conversion of Channel Maintenance Flows to Channel Structure Probabilities**.
- See **4. Final Results** including both a probability distribution and also a single expected value.

1. ERM Conceptual Diagram

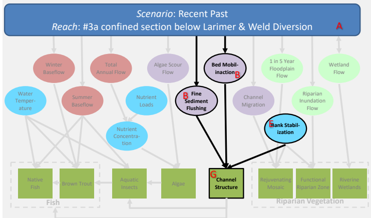

2. Narration

The channel structure computations are a function of the interaction of the hydrology of each flow scenario and the channel/sediment conditions in each reach. The goal of this process is to compute scores for both fine sediment flushing and bed mobility, then to collapse these scores into a probabilistic estimate of the channel structure falling into one of the following states (further described in Table II.9 of ERM Report [as shown below] and listed from lowest to highest) (1) Entrenched, (2) Largely immobile and homogeneous, (3) Partially mobile and diverse, and (4) Clean and diverse. These computations are outlined in the following steps:

A. The model is designated to perform computations on the Recent Past Scenario and on Reach: #3a a confined section below Larimer & Weld Diversion. Note that all computations below are demonstrated in the red highlighted box in the following table. All flow data consists of daily average discharge from 11/1/1949 to 10/31/2005. The Recent Past flow scenario used in this case study is based upon historic gage data. Note that the flow scenarios are derivations of this Recent Past Scenario.

B. The extent of both Fine Sediment Flushing and Bed Mobilization were computed using the Channel Maintenance Flow Index (CMFI) that was developed for this project (see ERM Appendix B1 for more details on this shear stress exceedance relationship which also incorporates duration).
   - While the two indices were computed independently, they can be thought of as a continuous channel-maintenance gradient from the lowest probability for Fine Sediment Flushing to the highest probability of Bed Mobilization. Flushing transitions to full bed Mobility when CMFI-F exceeds 1.0. The computed values for the Recent Past flow scenario result in a **CMFI-F = 1.12 and CMFI-M = 0.54.**

C. Next, the CMFI values were converted into a single mean probability of optimal Channel Condition (scaled from 0 to 1) using a value function (as seen in the graph in cells E3:F12 and also in Figure B1.5 of the ERM Appendix). **CMFI-F and CMFI-M scores are thus translated into an initial estimate of 52% probability of optimal Channel Structure in Reach 3a given the Recent Past Flow Scenario**. Additionally, a starting standard deviation was needed to define the spread of the distribution and thus a standard deviation of 0.25 was assumed for a mean value of 1.0 and reduced accordingly for lesser means.

D. A subsequent step in this analysis adjusts the uncertainty and level of this prediction based upon the frequency of adequate flood flows. **Due to the frequency of Bed Mobility inducing peak flows (return interval of 4.7 years for a $\tau_* > 0.030$ which is longer than the desired return interval (3 years)), the probability was adjusted down to 37% and the standard deviation set to 0.19.**

E. Bank stabilization (summarized in Table II.28 of the ERM Report and also reproduced below) is a rating of how much of the banks are rip-rapped, leveed, or otherwise not able to be moved or eroded. **Reach 3a is classified as protected**. Thus, Bank Stabilization is used to cap the upper limit mean of the Channel Structure probabilities. Reach 3a with its protected condition (capping the probability at 75%) was not engaged for this scenario and reach.

F. Finally, the mean and estimated standard deviations are turned into a normal distribution across the four states of Channel Structure. The first step in this process was to compute the cumulative probabilities.

G. Finally the discrete probabilities were computed for each state. **For this case study, there is a 26% probability of the Channel Structure being Entrenched, 50% largely immobile and homogeneous, 22% Partially mobile and diverse, and 2% Clean and diverse**. As a final step a single expected value was computed to represent the final probability distribution.

Table II.9: Description of four states of channel structure that depend on the combined status of flushing flows, coarse substrate mobilization, channel migration flows, and extent of armoring.

State	Description
Clean and diverse	Flushing and bed mobility flow functions intact, substrate clean on surface, interstitial space open, vegetation encroachment not advancing, channel has wide variety of depth, velocity, substrate combinations with morphologically diverse features such as side channels, chutes, bars owing to substantial removal of lateral armoring.
Partially mobile and diverse	All three flow functions at least partially intact, flushing occurs at least every few years, interstitial space open in high stress zones such as riffles, vegetation encroachment slowly advancing in low stress zones, habitat diversity flows may be intact but lateral armoring partially limiting channel complexity.
Largely immobile and homogeneous	Bed mobilization and/or channel migration flows not intact, flushing partially or not intact, vegetation encroachment likely advancing, river increasingly canal-like with homogeneous habitat until partially reset by an extreme event that overcomes riprap armoring in isolated locations, substrate flushing at least partially intact, habitat diversity flows could be intact but stabilization present.
Entrenched	Partial to no substrate cleaning, channel maintenance absent, interstitial space not opened > 3 to 5 years, extensive bank stabilization, canal-like homogeneous channel .

Table II.28: Classes of channel stabilization.

Class	Description -- based on length of stabilizing features relative to bank length (twice the channel length)
Minimal	Stabilized length occurring at any distance from channel center line is < 5% of bank length.
Altered	Stabilized length >5% of bank length and stabilized length occurring <50 m from channel center line is <15% of bank length.
Protected	Stabilized length occurring <50 m from channel center line is 15 to 30% of bank length.
Stabilized	Stabilized length occurring <50 m from channel center line is >30% of bank length.

Note: Current values for Reaches 3a, 3b, and 7 are Protected, Protected, and Altered, respectively.

Table numbers (II.9 and II.28) correspond to those in Shanahan et al. 2014

3. Conversion of Channel Maintenance Flow Index (CMFI) to Channel Structure Probabilities

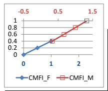

Value function of CMFI vs. Mean Prob of Channel

CMFI_F	CMFI_M	Mean
0	0	0
0.5	0	0.2
1	0.33	0.4
	0.67	0.6
	1	0.8
	1.33	1

	Return Interval Ranges		Weighting factors	
	RI_F	RI_M	*mean	*SD
	0	0	1.2	0.7
Min	1.30	2.00	1.2	0.7
Desired	1.50	3.00	1	1
Max	5.00	7.00	0.3	2
Weight if > MAX			0	3

Bank Stabilization	
Reach	Class
3a	3
3b	3
6	2
7	2

Class	Descriptio	Max Mean Prob
1	minimal	no change
2	altered	0.875
3	protected	0.75
4	stabilized	0.625

Thresholds between classes

0	0.25	0.5	0.75	1

Narration A		Narration B		Narration C	0.25		Narration D			
Scenario	Reach	CMFI Flushing t*c=0.021	CMFI Mobility t*c=0.030	(based on above Value Function) Mean	(multiple of mean) Standard Deviation	Cntrl'ng? Flushing or Mobility	Return Interval_F (yr)	Return Interval_M (yr)	Frequency Weight (Mean)	Frequency Weight (SD)
Reconstructed Native	3a	3.58	1.69	1.00	0.25	M	1.2	1.5	1.20	0.70
Reconstructed Native	3b	5.09	2.13	1.00	0.25	M	1.1	1.4	1.20	0.70
Reconstructed Native	7	6.49	6.10	1.00	0.25	M	1.0	1.1	1.20	0.70
Recent Past	**3a**	1.12	0.54	0.52	0.13	M	2.1	4.7	0.71	1.42
Recent Past	3b	1.83	0.70	0.62	0.15	M	1.6	3.5	0.91	1.13
Recent Past	7	2.14	1.77	1.00	0.25	M	1.4	2.1	1.19	0.71
Present Operations	3a	0.22	0.18	0.09	0.02	M	6.8	13.4	0.00	0.00
Present Operations	3b	0.56	0.19	0.23	0.06	M	3.8	12.6	0.00	0.00
Present Operations	7	1.62	0.51	0.50	0.13	M	2.7	5.2	0.62	1.55
Additional Water Develop	3a	0.08	0.07	0.03	0.01	M	25.1	39.9	0.00	0.00
Additional Water Develop	3b	0.14	0.08	0.06	0.01	M	16.8	35.1	0.00	0.00
Additional Water Develop	7	0.33	0.18	0.13	0.03	M	7.8	18.1	0.00	0.00
Driest Climate	3a	0.01	0.00	0.00	0.00	M	27.3	100.0	0.00	0.00
Driest Climate	3b	0.03	0.00	0.01	0.00	M	24.1	100.0	0.00	0.00
Driest Climate	7	0.14	0.02	0.05	0.01	M	9.7	22.4	0.00	0.00
HighBase-ModeratePeak	3a	0.17	0.00	0.07	0.02	M	1.3	100.0	0.00	0.00
HighBase-ModeratePeak	3b	0.90	0.00	0.36	0.09	M	1.0	100.0	0.00	0.00
HighBase-ModeratePeak	7	1.57	0.51	0.51	0.13	M	1.0	1.1	1.20	0.70
StableBase-HighPeak	3a	1.68	1.20	0.92	0.23	M	1.2	1.3	1.20	0.70
StableBase-HighPeak	3b	2.03	1.55	1.00	0.25	M	1.2	1.3	1.20	0.70
StableBase-HighPeak	7	1.24	3.18	1.00	0.25	M	1.2	1.3	1.20	0.70
DryBase-HighPeak	3a	1.75	1.37	1.00	0.25	M	1.2	1.3	1.20	0.70
DryBase-HighPeak	3b	1.92	1.70	1.00	0.25	M	1.2	1.3	1.20	0.70
DryBase-HighPeak	7	1.32	3.64	1.00	0.25	M	1.2	1.3	1.20	0.70
StableBase-LowPeak	3a	0.0	0.0	0.00	0.00	M	100.0	100.0	0.00	0.00
StableBase-LowPeak	3b	0.0	0.0	0.00	0.00	M	100.0	100.0	0.00	0.00
StableBase-LowPeak	7	0.9	0.0	0.34	0.09	M	1.3	100.0	0.00	0.00

		Narration D (cont.) over 1.0		Narration E		Narration F Cumulative Probabilities					
Scenario	Reach	Weighted Mean	Weighted Standard Deviation	Bank Stabilization	Stabilization Weighted Mean	Below 0	Armored and clogged	Armored but flushed	Partially Open	Open and Diverse	Above 1
Reconstructed Native	3a	1.20	0.18	3	0.75	0.000	0.002	0.078	0.500	0.922	0.078
Reconstructed Native	3b	1.20	0.18	3	0.75	0.000	0.002	0.078	0.500	0.922	0.078
Reconstructed Native	7	1.20	0.18	2	0.88	0.000	0.000	0.017	0.239	0.761	0.239
Recent Past	**3a**	0.37	0.19	3	0.37	0.023	0.258	0.754	0.979	1.000	0.000
Recent Past	3b	0.56	0.17	3	0.56	0.001	0.037	0.360	0.859	0.994	0.006
Recent Past	7	1.19	0.18	2	0.88	0.000	0.000	0.018	0.243	0.757	0.243
Present Operations	3a	0.00	0.00	3	0.00	0.500	1.000	1.000	1.000	1.000	0.000
Present Operations	3b	0.00	0.00	3	0.00	0.500	1.000	1.000	1.000	1.000	0.000
Present Operations	7	0.31	0.20	2	0.31	0.056	0.377	0.833	0.988	1.000	0.000
Additional Water Develop	3a	0.00	0.00	3	0.00	0.500	1.000	1.000	1.000	1.000	0.000
Additional Water Develop	3b	0.00	0.00	3	0.00	0.500	1.000	1.000	1.000	1.000	0.000
Additional Water Develop	7	0.00	0.00	2	0.00	0.500	1.000	1.000	1.000	1.000	0.000
Driest Climate	3a	0.00	0.00	3	0.00	0.500	1.000	1.000	1.000	1.000	0.000
Driest Climate	3b	0.00	0.00	3	0.00	0.500	1.000	1.000	1.000	1.000	0.000
Driest Climate	7	0.00	0.00	2	0.00	0.500	1.000	1.000	1.000	1.000	0.000
HighBase-ModeratePeak	3a	0.00	0.00	3	0.00	0.500	1.000	1.000	1.000	1.000	0.000
HighBase-ModeratePeak	3b	0.00	0.00	3	0.00	0.500	1.000	1.000	1.000	1.000	0.000
HighBase-ModeratePeak	7	0.61	0.09	2	0.61	0.000	0.000	0.114	0.943	1.000	0.000
StableBase-HighPeak	3a	1.10	0.16	3	0.75	0.000	0.001	0.061	0.500	0.939	0.061
StableBase-HighPeak	3b	1.20	0.18	3	0.75	0.000	0.002	0.078	0.500	0.922	0.078
StableBase-HighPeak	7	1.20	0.18	2	0.88	0.000	0.000	0.017	0.239	0.761	0.239
DryBase-HighPeak	3a	1.20	0.18	3	0.75	0.000	0.002	0.078	0.500	0.922	0.078
DryBase-HighPeak	3b	1.20	0.18	3	0.75	0.000	0.002	0.078	0.500	0.922	0.078
DryBase-HighPeak	7	1.20	0.18	2	0.88	0.000	0.000	0.017	0.239	0.761	0.239
StableBase-LowPeak	3a	0.00	0.00	3	0.00	0.500	1.000	1.000	1.000	1.000	0.000
StableBase-LowPeak	3b	0.00	0.00	3	0.00	0.500	1.000	1.000	1.000	1.000	0.000
StableBase-LowPeak	7	0.00	0.00	2	0.00	0.500	1.000	1.000	1.000	1.000	0.000

4. Final Results

		Narration G Final probabilities for each state				
Scenario	Reach	Entrenched	Largely immobile and homogeneous	Partially mobile and diverse	Clean and diverse	sum (check)
Reconstructed Native	3a	0.002	0.075	0.422	0.500	1.000
Reconstructed Native	3b	0.002	0.075	0.422	0.500	[illegible]
Reconstructed Native	7	0.000	0.016	0.222	0.761	[illegible]
Recent Past	**3a**	0.258	0.497	0.224	0.021	1.000
Recent Past	3b	0.037	0.323	0.499	0.141	1.000
Recent Past	7	0.000	0.018	0.225	0.757	1.000
Present Operations	3a	1.000	0.000	0.000	0.000	1.000
Present Operations	3b	1.000	0.000	0.000	0.000	1.000
Present Operations	7	0.377	0.456	0.155	0.012	1.000
Additional Water Develop	3a	1.000	0.000	0.000	0.000	1.000
Additional Water Develop	3b	1.000	0.000	0.000	0.000	1.000
Additional Water Develop	7	1.000	0.000	0.000	0.000	1.000
Driest Climate	3a	1.000	0.000	0.000	0.000	1.000
Driest Climate	3b	1.000	0.000	0.000	0.000	1.000
Driest Climate	7	1.000	0.000	0.000	0.000	1.000
HighBase-ModeratePeak	3a	1.000	0.000	0.000	0.000	1.000
HighBase-ModeratePeak	3b	1.000	0.000	0.000	0.000	1.000
HighBase-ModeratePeak	7	0.000	0.114	0.829	0.057	1.000
StableBase-HighPeak	3a	0.001	0.060	0.439	0.500	1.000
StableBase-HighPeak	3b	0.002	0.075	0.422	0.500	1.000
StableBase-HighPeak	7	0.000	0.016	0.222	0.761	1.000
DryBase-HighPeak	3a	0.002	0.075	0.422	0.500	1.000
DryBase-HighPeak	3b	0.002	0.075	0.422	0.500	1.000
DryBase-HighPeak	7	0.000	0.016	0.222	0.761	1.000
StableBase-LowPeak	3a	1.000	0.000	0.000	0.000	1.000
StableBase-LowPeak	3b	1.000	0.000	0.000	0.000	1.000
StableBase-LowPeak	7	1.000	0.000	0.000	0.000	1.000

Full Distribution

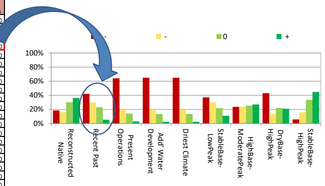

Single Expected Value, reported in Figure 6 of manuscript

State	Entrenched	Largely immol	Partially mobi	Clean and diverse
Prob for each state	0.5626	0.0006	0.0365	0.3598
Scaling parameters	0.000	0.333	0.666	1.000
Partial probabilities	0.000	0.000	0.024	0.360
**Expected Value**				**0.384**
